# Supplementary material for: Development of a micro-combined heat and power powered by an opposed-piston engine in building applications
Source: Nat Commun. 2024 May 23;15:4404. doi: 10.1038/s41467-024-48627-0 (PMC11116428; doi:10.1038/s41467-024-48627-0)
Supplement: Supplementary file 1 — Supplementary Information [file 41467_2024_48627_MOESM1_ESM.pdf]

Supplementary materials:

Development of a Micro-Combined Heat and Power Powered by an Opposed-Piston Engine in Building Applications

Zhiming Gao[1*], Philip Zoldak[2], Jacques Beaudry-Losique[2], Tony Mannarino[2], Jonathan Mansinger[2], Maysam Molana[2], Mingkan Zhang[1], Praveen Cheekatamarla[1], Ahmed Abuheiba[1], Hailin Li[3], Brian Fricke[1] and Kashif Nawaz[1]

[1]Oak Ridge National Laboratory, Oak Ridge, TN 37831

[2]Enginuity Power Systems, 730 S Washington St, Alexandria, VA 22314

[3]West Virginia University, Morgantown, WV 26506-6106

*Corresponding authors

Email: gaoz@ornl.gov; Tel:+1-865-241-5018

Supplemental Note 1: Summary of mCHP technologies available

The major micro–combined heat and power (mCHP) technologies include internal combustion engines (ICEs), micro gas turbines, micro-Rankine cycles, Stirling engines, and thermophotovoltaic generators [3, 18–28]. Table S1 summarizes these representative technologies available in the public domain. Clearly, their performance is substantially different. This considerable variation in performance is a direct result of employing different mCHP technologies, design and fabrication approaches, and system integration and optimization considerations, all while considering cost-effectiveness. Additionally, specific procedures and standards in different countries may also contribute to these differences.

Table S1. Summary and characteristics of mCHPs for single-family houses and small buildings

**Tech.**	**OEM/ academic**	**Model**	**Electric power (kW)**	**Heat power (kW)**	**Electrical efficiency (%)**	**CHP efficiency (%)**	**Ref.**
ICE (4)	EC Power	XRGI 6	3.0–6.0	8.1–13.70	24.8–30.1	81.1–87.1	[1]
ICE (4)	EC Power	XRGI 9	4.5–9.0	12.0–21.3	25.9–30.4	84.0–91.9	[1]
ICE (4)	Honda	Ecowill	1	2.8–3	20.0–23.5	74.5–85.0	[2, 3]
ICE (4)	Marathon Engine System	Ecopower	1.0–4.7	—	24.4–25	65.0–94.5	[4,5, 6,7]
ICE (4)	Senertec	Dachs	5.0–5.5	12.5–15.5	24.0–27.7	61.0–92.0	[2,4,6, 8]
ICE (4)	Totem	Totem 10	5.0–10.0	14.5–21.6	23.6–29.7	90.2–93.8	[9]
ICE (4)	Yanmar	CP5WN	5.0	10.0	28.0	84.0	[10]
ICE (4)	Yanmar	CP10WN	10.0	17.0	31.5	85.0	[10]
Turbine	Academic	Prototype	1.5	4.7	6.3	—	[11]
Turbine	MTT	Prototype	3	15	16	96.0	[2]
Stirling	Stirling Denmark	SM5A	9	25	20.8	84.5	[6]
Stirling	Solo	Solo 161	2–9.5	8–26	20–26.8	72–96	[2,4,6, 12]
Stirling	Whisper Tech	—	0.75–1	4.9–6.5	6–12	80–82	[4,13]
ORC	Academic	Prototype	0.9	47.3	1.41	78.7	[14]
ORC	COGEN Microsystems	Prototype	2.5	11	18.5	87.2	[2]
ORC	Energetix	Genlec	1	8	10	90	[2]
TPV	Academic	Prototype	0.3	8.5	1.3–2	83–84	[15]
TPV	JX Crystal	Prototype	1.5	9.4	12.3	92.1	[2]
Fuel cell	REDOX	Redox Cube	25	—	54.0	—	[16]
Fuel cell	WATT Fuel Cell	WATT Imperium	0.5–1.15	—	—	—	[17]

Note: CHP = combined heat and power; ORC = organic Rankine cycle; TPV = thermophotovoltaic.

Supplemental Note 2: Ten testing cases for evaluating the mCHP

Six cases were tested under stoichiometric combustion modes with the air–fuel ratio set to λ = ~1.0. These cases are listed in Table S2. The first three cases measured the electrical efficiency and overall mCHP thermal efficiency with waste heat recovered and stored in the water tank at various electric power generation ratings. The fourth, fifth, and sixth cases measured the electrical efficiency and overall mCHP thermal efficiency with external thermal demand hot water supply and space heating at different electric power generation capacities.

Table S2. Six testing cases for evaluating the mCHP under stoichiometric combustion modes

Case	Description
1	3.5 kW electricity with waste heat recovered and stored in the water tank; the water tank is heated from 26.7 °C to 60 °C without external heat demand
2	4.5 kW electricity with waste heat recovered and stored in the water tank; the water tank is heated from 26.7 °C to 60 °C without external heat demand
3	7.5 kW electricity with waste heat recovered and stored in the water tank; the water tank is heated from 61.1 °C to 71.1 °C without external heat demand
4	3.5 kW electricity with external thermal demand for space heating; the water tank temperature starts at 40 °C
5	4.5 kW electricity with external thermal demand for space heating; the water tank temperature starts at 40 °C
6	6.0 kW electricity with external thermal demand for space heating; the water tank temperature starts at 40 °C

Four cases were tested under lean combustion modes, shown in Table S3. The fourth case aims to repeat the third case and was used to calculate the uncertainty associated with the measurements. All the lean cases recovered waste heat and stored it in the tank. In all the lean combustion cases, the control module used the lambda sensor and intake port actuator to maintain a 30% excess of air consumption or λ = ~1.3, and the ignition timing for all lean modes was advanced approximately 10 °CA to maximize torque operation compared with the stoichiometric modes.

Table S3. Four testing cases for evaluating the mCHP under lean combustion modes

Case	Description
7	3.5 kW electricity with waste heat recovered and stored in the water tank; the water tank is heated from 26.7 °C to 60 °C without external heat demand
8	4.5 kW electricity with waste heat recovered and stored in the water tank; the water tank is heated from 26.7 °C to 60 °C without external heat demand
9	6.0 kW electricity with waste heat recovered and stored in the water tank; the water tank is heated from 26.7 °C to 60 °C without external heat demand
10	6.0 kW electricity with external thermal demand for hot water supply and space heating; the water tank is heated from 26.7 °C to 60 °C without external heat demand

In compliance with ASHRAE standards, water heaters are required to supply hot water at a temperature of 60 °C (140 °F). Therefore, the cases 1–2 and 7–10 are designed to raise water temperature from 26.7 °C to 60 °C without external heat load demand. In the case 3, the water within the tank is heated from 61.1 °C to 71.1 °C without external heat demand. Additionally, the cases 4–6 are designed because typical US domestic space heating temperature, generated from heat pumps and residential furnaces, is in the range of 21.1 °C to 51.7 °C (70 °F to 125 °F). These

cases have the water tank beginning at 40 °C and incorporating external thermal demand for space heating requirements.

Supplemental Note 3: Measurement uncertainties

The propagation of the uncertainty method [18] was used to calculate the uncertainty associated with the measurements in the work. The method estimates the uncertainty in a parameter from the uncertainties in the measurements used to calculate the parameter. For example, the uncertainty of energy in the burnt fuel depends on the uncertainties of fuel flow measurement and lower heating value (LHV) calculation in the testing cases. In the same manner, the uncertainty of total efficiency of the system is a function of measurement uncertainties in temperature, pressure, and mass flow rates. The key instruments involved in the measurements for the calculation of mCHP efficiencies are listed in Table S4. In the current system, the uncertainties of electrical, thermal, and total efficiency are derived as 3.28%, 2.26%, and 2.51%, respectively. All the experimental uncertainty has been shown in Figure 4 in the revised manuscript.

Table S4. Measurement range and sensitivity of instruments used in the tests.

Instrument description	Measurement range	Sensitivity
K-type thermocouple	−200 °C to 1250 °C	2.2 °C
Pressure transducer	Different ranges for different applications, such as in-cylinder, oil, water, and manifold	2.1%
Coolant flow meter	0.0–6.6 GPM	0.8% of the measured value +0.5% of the final value of the range
Gas flow meter	1–120 SFCM	1%
Voltage transducer	0–500 V	1%
Current transducer	0–300 A	1%
Oxygen sensor	20–900 mV	1–45 ppm

Supplemental Note 4: The sensitivity comparison of the repeated tests

Regarding test repeatability and reproducibility, the authors repeated the test for the 5.93 AC kW of the lean combustion modes (i.e., the cases 9 and 10). The repeatability results are shown in Table S5. The results from Figure S1 reveal that the maximum sensitivity is less than 1.8% for all the performing efficiencies; power; opposed-piston four-stroke (OP4S) exhaust; and coolant. It indicates that the mCHP is capable of excellent repeatability and reproducibility.

Table S5. Repeatability and reproducibility results for the mCHP.

Parameter / Case	Waste heat recovered and stored in the tank		Repeatability resolution
	9	10	(Based on case 9)
AC electrical power (kW)	5.93	5.98	1.8%
Engine brake energy efficiency (%)	39.1	38.7	−1.0%
OP4S-out coolant temperature (°C)	79.0	79.4	0.5%
OP4S exhaust temperature (°C)	692	699	1.0%
AC electrical power efficiency (%)	35.2	34.8	−1.1%
DC electrical power efficiency (%)	33.8	33.4	−1.2%
Overall mCHP efficiency (%)	93.2	93.6	0.4%

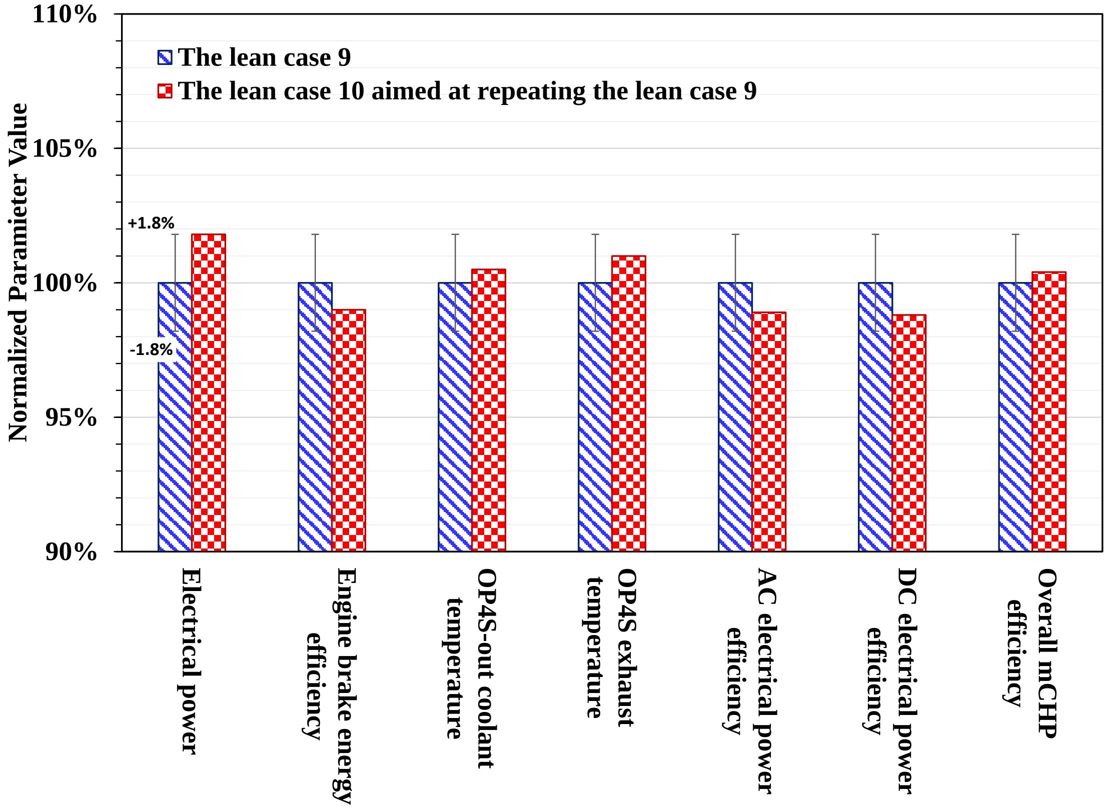

Figure S1. Repeatability resolution analysis for cases 9 and 10. Note: the error bar shows the maximum sensitivity of mCHP test repeatability and reproducibility.

Supplemental Note 5: Fuel composition

Table S6. Fuel composition of natural gas used in the testing cases shown in Figure 4.

Nitrogen	0.1953%
Methane	91.7073%
Ethane	7.4238%
Propane	0.2544%
Butane	0.0178%
Isobutane	0.0208%
Pentane	0.0012%
Isopentane	0.0036%
Hexanes	0.0029%
Others	0.3729%
Lower heating value (kJ/mol)	848
Higher heating value (kJ/mol)	939
Carbon	75.7%
Molecule weight (g/mol)	17.0

Supplemental Note 6: The electrical Performance of the mCHP system

For the stoichiometric combustion modes, Table S7 shows the electrical power outputs of the mCHP with peak AC electricity efficiency of 26.4%. The power outputs are in the range of 3.33–7.70 AC kW (i.e., 3.20–7.39 DC kW) for the six selected cases. The AC electricity efficiencies vary between 16.8% and 26.4%, and the DC electricity efficiencies vary between 16.1% and 25.3%. The results reveal that larger electric power output leads to higher electrical efficiency, whether waste heat is recovered and stored in the water tank or external thermal load is applied for hot water supply and space heating. This expected result occurs because engine throttling loss is usually reduced with higher engine power.

Table S7. Electrical efficiency of the mCHP prototype operating under the stoichiometric modes.

**Case / Parameter**	**Waste heat recovered and stored in the tank**			**Thermal load for hot water supply and space heating**		
	**1**	**2**	**3**	**4**	**5**	**6**
Engine speed (rpm)	2,877	2,900	3,050	2,776	2,918	2,883
AC electrical power (kW)	3.89	4.60	7.70	3.33	4.74	6.18
DC electrical power (kW)	3.74	4.42	7.39	3.19	4.55	5.94
Engine brake energy efficiency (%)	19.1	19.9	29.2	18.6	20.5	28.4
DC electrical power efficiency (%)	16.5	17.2	25.3	16.1	17.7	24.5
AC electrical power efficiency (%)	17.2	17.9	26.4	16.8	18.4	25.5

For the lean combustion modes, Table S8 shows the electrical power output of the mCHP with peak AC electricity efficiency of 35.2%. Power outputs are in the range of 3.64–5.98 AC kW (i.e.,

3.5–5.8 DC kW) for the four tested cases. The AC electricity efficiencies vary between 22.7% and 35.2%, and the DC electricity efficiencies vary between 21.8% and 33.8%.

Table S8. Electrical efficiency performance of the mCHP prototype in lean combustion mode.

**Case Parameter**	**Waste heat recovered and stored in the tank**			
	**7**	**8**	**9**	**10***
Engine speed (rpm)	2,705	2,780	2,850	2,904
AC electrical power (kW)	3.64	4.62	5.93	5.98
DC electrical power (kW)	3.50	4.43	5.70	5.74
Engine brake energy efficiency (%)	25.2	32.4	39.1	38.7
AC electrical power efficiency (%)	22.7	29.2	35.2	34.8
DC electrical power efficiency (%)	21.8	28.0	33.8	33.4

** Case 10 aims to repeat case 9.*

Supplemental Note 7: The Thermal Performance of the mCHP system

Table S9. Thermal energy performance of the mCHP prototype under the stoichiometric modes.

**Case Parameter**	**Waste heat recovered and stored in the tank**			**Thermal load for hot water supply and space heating**		
	**1**	**2**	**3**	**4**	**5**	**6**
Exhaust temperature at entrance of water tank (°C)	823.6	822.6	777.9	813.8	844.7	763.9
Exhaust temperature at exit of water tank (°C)	31.3	38.0	149.9	77.9	36.6	138.3
Coolant temperature at entrance of engine (°C)	65.2	66.2	77.8	73.7	70.1	72.7
Coolant temperature at exit of engine (°C)	70.6	71.7	83.2	79.7	76.8	78.7
Exhaust waste recovery (kW)	8.40	9.20	6.73	6.90	9.50	5.53
Coolant waste recovery (kW)	11.72	12.42	12.24	8.14	12.03	10.29
Waste heat available from exhaust and coolant (kW)	20.75	21.62	18.97	15.24	21.53	15.82
Waste heat recovery efficiency (%)	97.0	95.5	81.1	85.9	95.8	81.7
Overall mCHP efficiency with AC output (%)	93.23	92.19	82.13	84.21	92.34	82.08
Overall mCHP efficiency with DC output (%)	92.63	91.55	81.19	82.73	91.67	81.16

Table S10. Thermal energy performance of the mCHP prototype in the lean combustion mode.

Case Parameter	Waste heat recovered and stored in the tank			
	**7**	**8**	**9**	**10***
Exhaust temperature at entrance of water tank (°C)	747.6	698.3	692.1	699.6
Exhaust temperature at exit of water tank (°C)	46.7	44.6	47.7	43.4
Coolant temperature at entrance of engine (°C)	74.1	74.2	74.4	73.8
Coolant temperature at exit of engine (°C)	79.1	79.2	79.0	79.4
Exhaust waste recovery (kW)	5.78	5.43	5.52	6.06
Coolant waste recovery (kW)	6.24	5.47	4.69	4.77
Waste heat available from exhaust and coolant (kW)	13.42	12.18	11.77	12.08
Waste heat recovery efficiency (%)	89.6	89.5	86.7	89.6
Overall mCHP efficiency with AC output (%)	87.96	88.47	86.40	88.37
Overall mCHP efficiency with DC output (%)	87.13	87.41	85.13	87.12

** Case 10 aims to repeat case 9.*

Supplemental Note 8: Comparison of engine efficiency between the opposed-piston engine and Cummins Westport engine

Figure S2 compares engine brake thermal energy efficiency between the opposed-piston engine and Cummins Westport natural gas engine as a function of engine power outputs. The Cummins Westport natural gas engine is a 2018 6.7 L, four-cycle, spark-ignition, in-line six-cylinder engine [19].

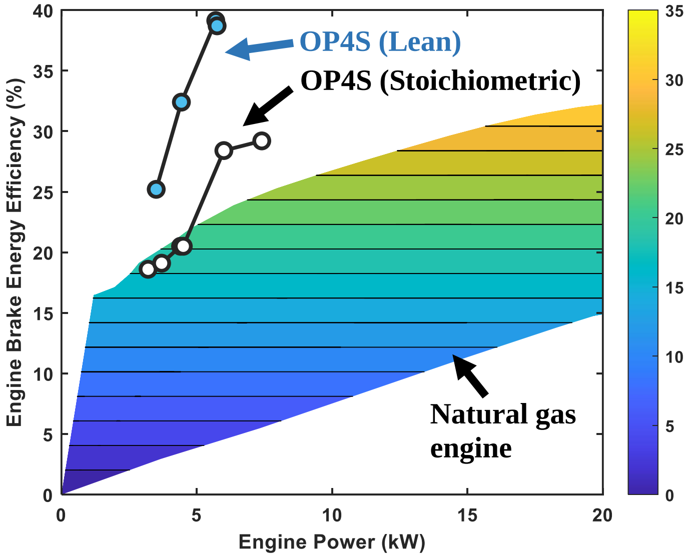

Figure S2. Comparison of engine brake energy efficiency for the OP4S and commercial natural gas engine as a function of engine power outputs (<20 kW). The commercial natural gas engine was operated in the stoichiometric mode. The colour bar shows the natural gas engine brake thermal energy efficiency.

Supplemental Note 9: The mCHP performance of case 1

Figure S3 shows an example of the mCHP prototype under testing conditions of case 1 (i.e., 3.89 AC kW or 3.74 DC kW), in which waste heat was recovered and stored in the water tank. In this case, the water tank was heated from 26.7 °C to 60 °C. The water temperature at the upper location was heated, but the bottom location remained at 26.6 °C. Significant temperature stratification occurred in the water tank. This result was confirmed by performing high-fidelity computational fluid dynamics simulations via Ansys Fluent commercial software. The simulation results for the waste heat recovery component are shown in Supplemental Note 10.

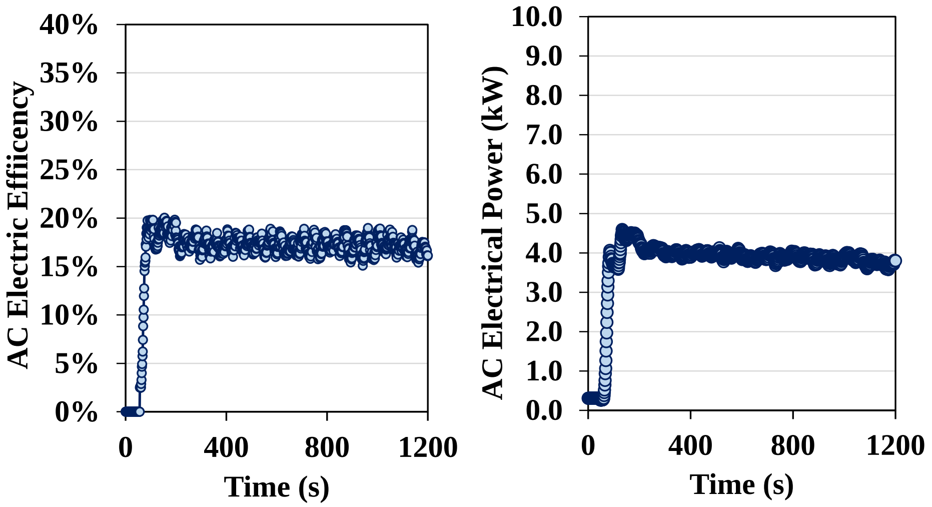

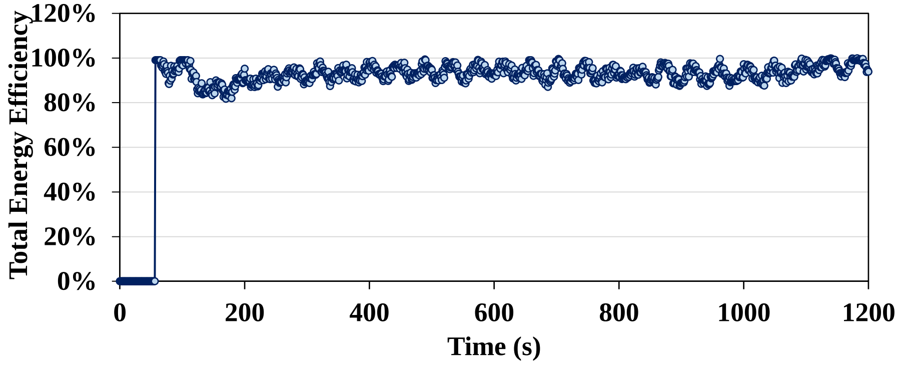

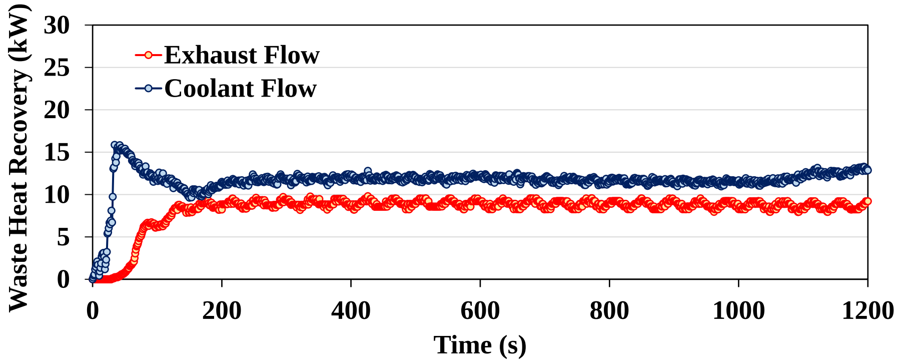

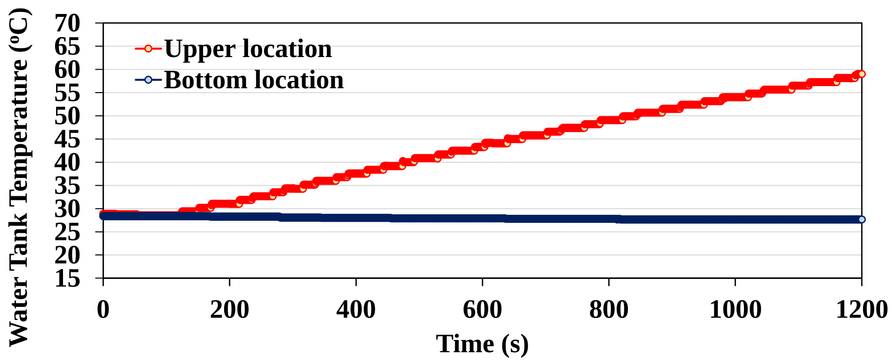

Figure S3. The mCHP prototype performance under testing conditions of 3.89 AC kW (i.e., 3.74 DC kW) and with waste heat recovered and stored in the water tank.

Supplemental Note 10: High-fidelity computational fluid dynamics simulations for the waste heat recovery component

The simulation results were confirmed by performing high-fidelity computational fluid dynamics (CFD) simulations via Ansys Fluent commercial software. The results show the water tank's status at 960 s. Validation of the CFD simulation is shown in Figure S4(b). The objectives of the CFD simulation were to expand the understanding of the performance of water heating and to support the optimal coil design for water heating in the future.

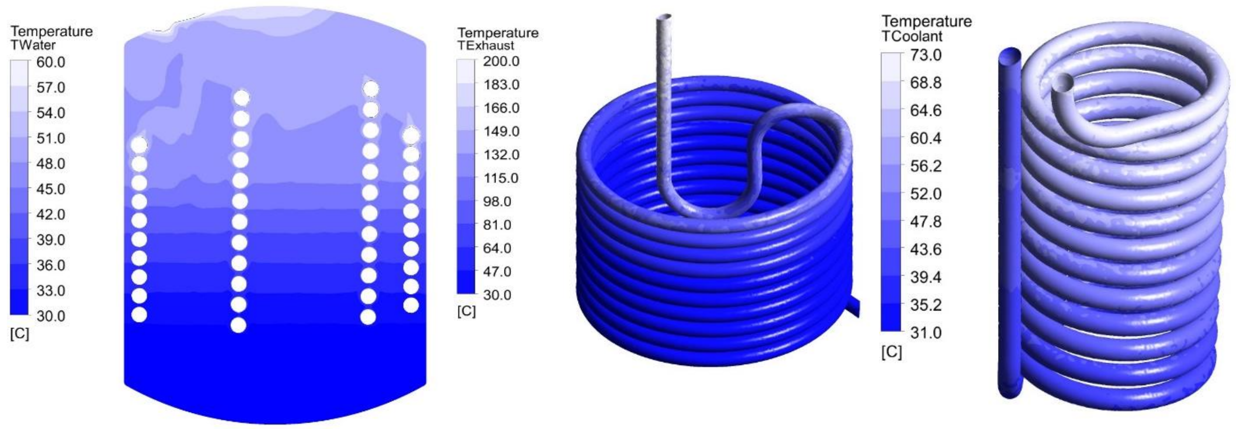

(a)

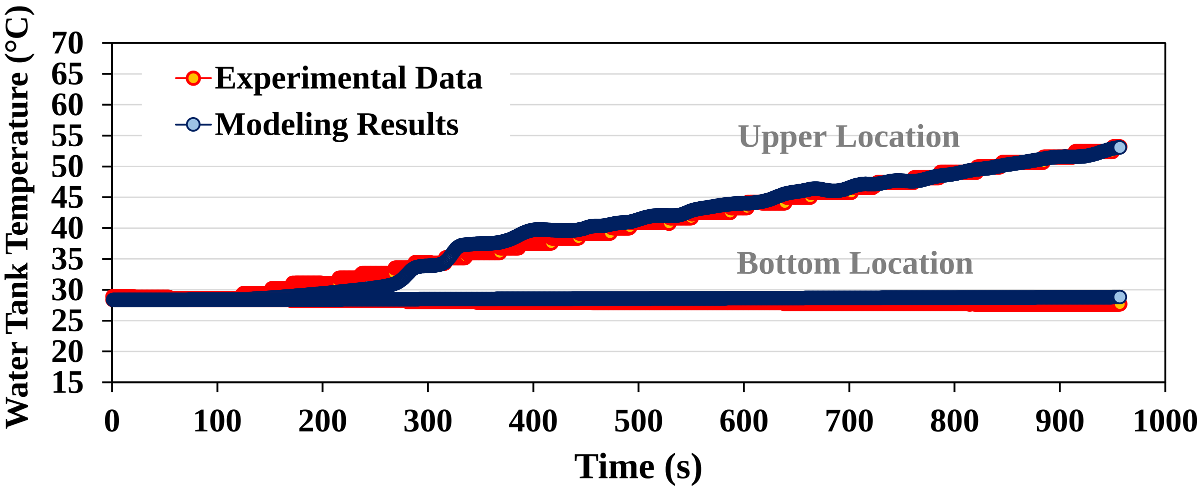

(b)

Figure S4. (a) High-fidelity CFD simulation of the mCHP waste heat recovery component; (b) validation of the CFD simulation based on the cases shown in Figure S4(a).

Supplemental Note 11: The mCHP performance of case 5

Figure S5 shows an example of the mCHP prototype under the testing conditions of case 5 (i.e., 4.74 AC kW or 4.55 DC kW), which has an external thermal load for hot water supply and space heating. In this case, the recovered heat was used continuously for space heating and hot water supply, while the water temperature was maintained at 40 °C. The air temperature increased from 26.4 °C to 37.7 °C, the hot water supply temperature was 38.8 °C, and the returning temperature was 27.7 °C.

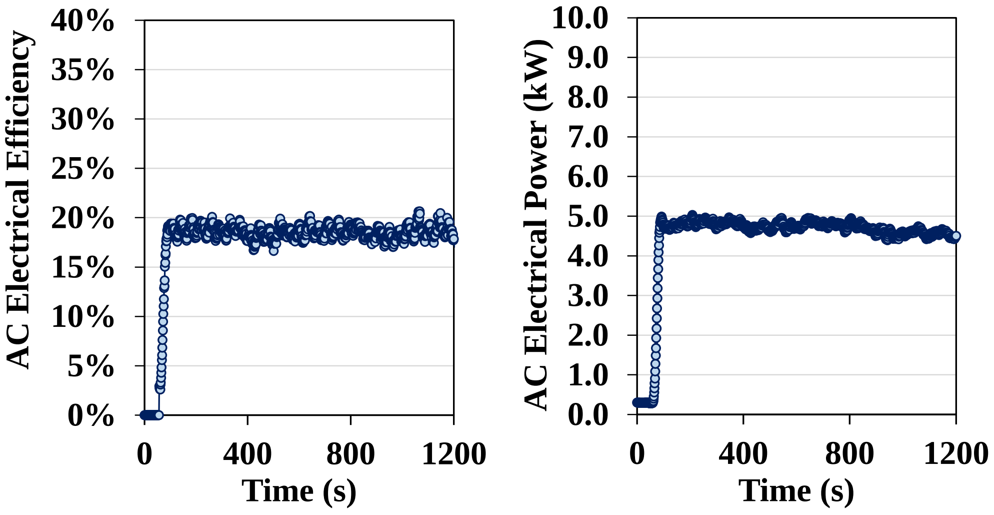

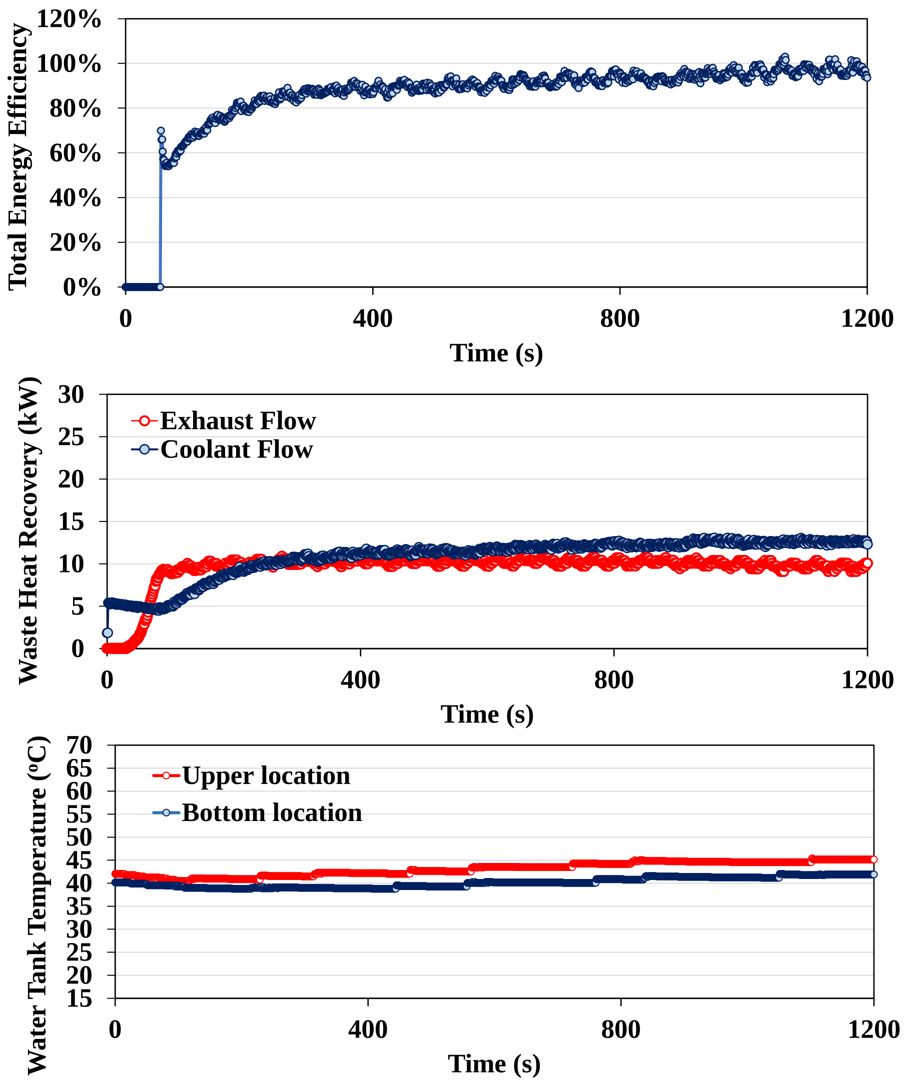

Figure S5. The mCHP prototype performance under the testing conditions of 4.74 AC kW (i.e., 4.55 DC kW) and with an external thermal load for hot water supply and space heating.

Supplemental Note 12: The mCHP performance of case 9

Figure S6 shows the mCHP prototype performance under the testing conditions of 5.93 AC kW (i.e., 5.7 DC kW) under the lean combustion mode (Case 9) and with waste heat recovered and stored in the water tank.

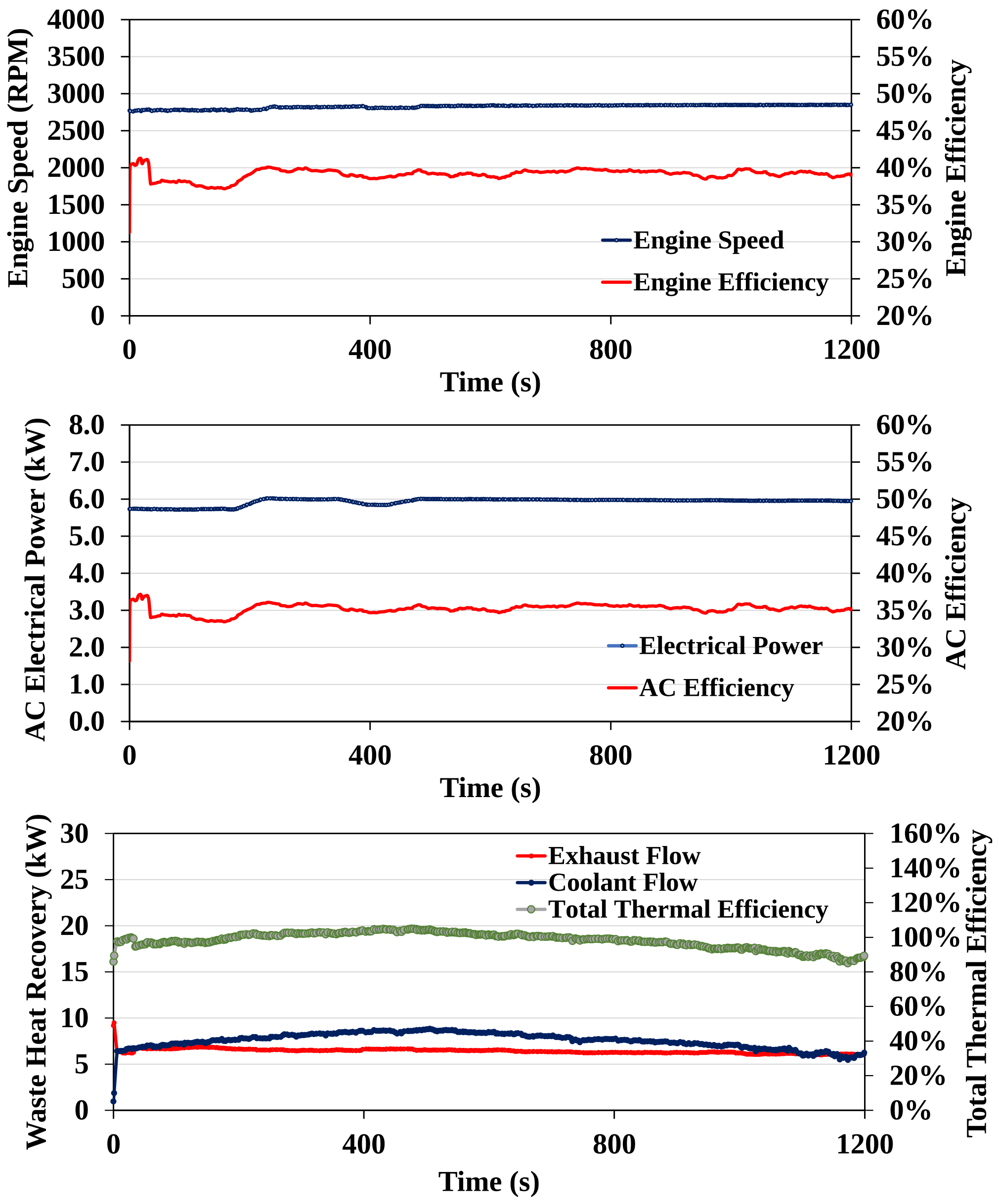

Figure S6. The mCHP prototype performance under testing conditions of 5.93 AC kW (i.e., 5.7 DC kW) under the lean combustion mode and with waste heat recovered and stored in the water tank.

Supplemental Note 13: Lean combustions modes cause higher latent heat loss

Two factors result in lower overall mCHP efficiencies in lean combustion modes. First, lean combustion modes lead to exhaust temperatures at the water tank exit ranging from 43.4 °C to 47.7 °C, indicating potential for improvement in the thermal energy control system for enhanced waste heat recovery. Second, lean combustion modes inherently lead to a lower ratio of moisture to dry air, increasing uncondensed water in the exhaust flow rejected to ambient conditions and causing higher latent heat loss. In the studies, the latent heat loss due to uncondensed water vapor in lean modes is 5.3%–~6.9% of HHV fuel energy. The loss in the stoichiometric modes is 2.2%–2.8% of HHV fuel energy with the exhaust temperature at the exit of water tank below 40 °C, but the loss is 9.2% of HHV fuel energy in the stoichiometric modes with the exhaust temperature at the exit of the water tank above 70 °C. The observation from Figure S7 illustrates that there is no condensate in stoichiometric modes if the exhaust temperature at the exit of the water tank is above 60 °C. That means all latent heat is lost to ambient condition.

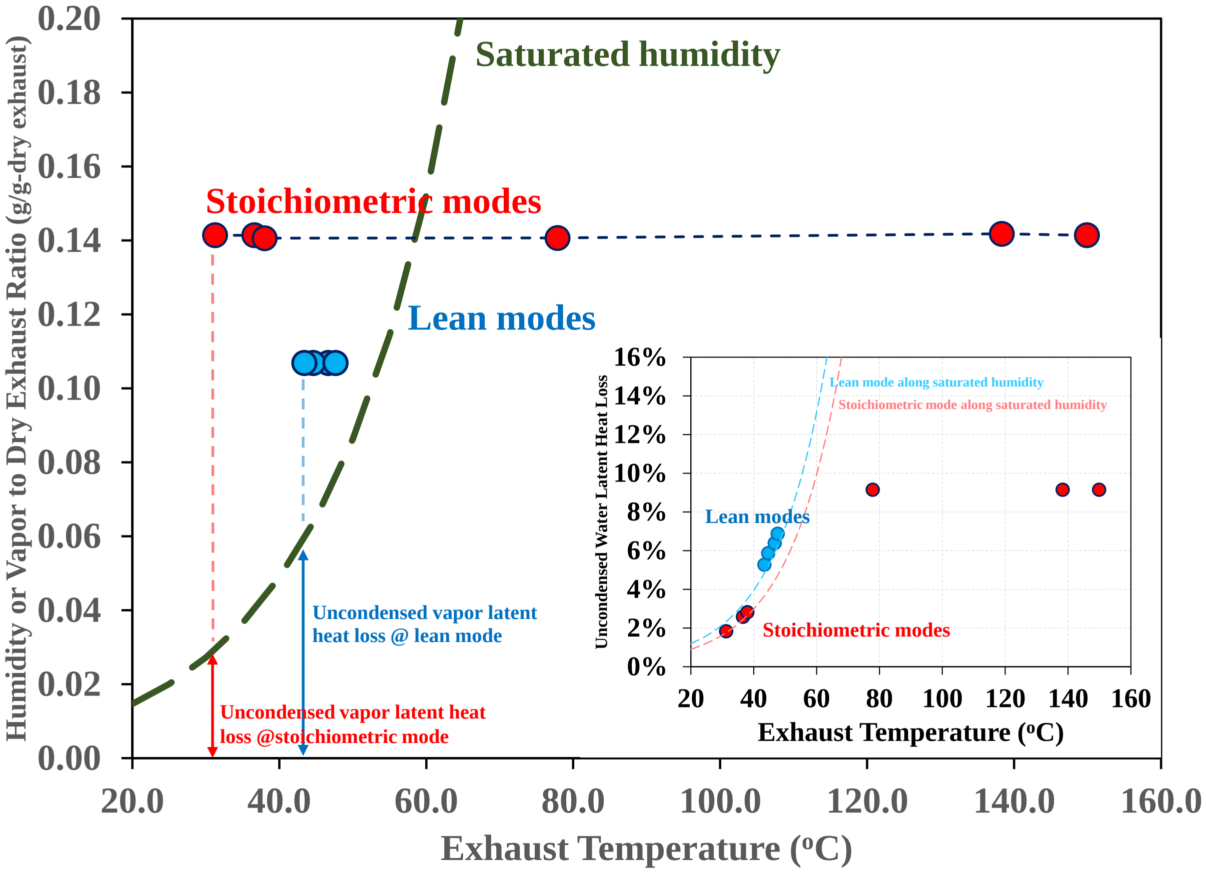

Figure S7. Comparison of latent heat loss due to uncondensed water vapor between lean and stoichiometric modes. In the embedded figure, the percentage of the uncondensed water latent heat loss is based on HHV fuel energy. Note: red circles are stoichiometric modes, and blue circles are lean modes.

Supplemental Note 14: The emissions of the mCHP system

The emissions, including CO, HC, and NO_x, were also measured from all the lean and stoichiometric modes. The results are shown in Figure S8, which reveals that the prototype using natural gas meets US EPA new source performance standards (NSPSs) for emissions for spark-ignition stationary engines used in the power generation of less than 19kW. For engine displacement of the mCHP, the NSPS emission standards require CO emissions of no more than 610 g/kWh and HC+NO_x emissions of no more than 8 g/kWh.

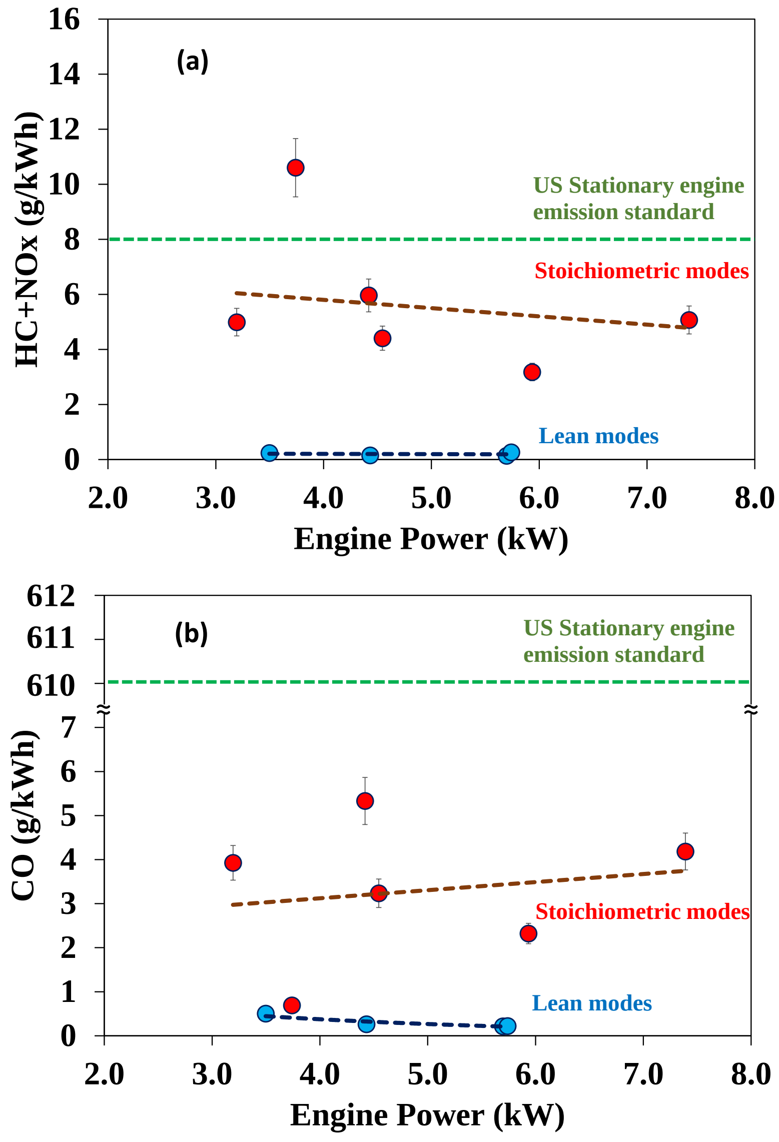

Figure S8. (a) HC+NO_x emissions of all the lean and stoichiometric modes as a function of engine power and (b) CO emissions of all the lean and stoichiometric modes as a function of engine power. The error bars of HC+NO_x and CO emissions shown are 10%. Note: red circles are stoichiometric modes, and blue circles are lean modes.

Supplemental Note 15: Exergy analysis

- **Methodology of Exergy Transfer and Destruction**

In the exergy analysis, effectiveness of the mCHP system is evaluated based on the maximum theoretical work defined as exergy relative to the standard environment state. The temperature and pressure of the reference environment were set as $T_0 = 298$ K and $p_0 = 1$ atm, and the exergy reference environmental air was assumed to be 75.67% N_2, 20.35% O_2, 3.12% H_2O, 0.03% CO_2, and 0.83% other species [20, 21].

By assuming a steady-state condition ignoring kinetic and potential energy, the exergy rate equation for each component in the mCHP (i.e., engine, generator, waste heat recovery component, and other components) can be calculated with the following equation:

$$\sum_j \left(1 - \frac{T_0}{T_j}\right) \dot{Q}_j + \dot{W}_{in} - \dot{W}_{out} + \sum \dot{E}_{in} - \sum \dot{E}_{out} - \dot{E}_d = 0 \quad \text{(S1)}$$

where $\dot{Q}_j$ is the heat transfer rate at the boundary of a given component; T_j is the temperature at the boundary where heat transfer occurs; T_0 is the reference temperature; $\dot{W}$ is the rate of work delivered including mechanical work and electrical work; $\dot{E}_{in}$ and $\dot{E}_{out}$ are the rates of exergy transfer into and out of a given component, respectively, owing to mass transfer into and out of the component; and $\dot{E}_d$ is the destructed exergy during the irreversible process.

For the engine component, the analysis assumed that the intake air was introduced at the same condition as that of the reference environment. Therefore, the intake air exergy was ignored in the study. The fuel exergy, $\bar{e}^{ch}_{fuel}$, was calculated based on the equation below:

$$\dot{E}_{fuel} = \dot{m}_{fuel} \bar{e}^{ch}_{fuel} = \dot{m}_{fuel} \sum_{i=1}^{3} y_i \bar{e}^{ch}_i \quad \text{(S2)}$$

where $\dot{m}_{fuel}$ is the fuel model rate, and y_i and $\bar{e}^{ch}_i$ represent the mole fraction of each species and the standard chemical exergy of each species. For natural gas, three main components—CH_4, C_2H_6, and C_3H_8—are considered and listed in Table S11.

Table S11. Specific chemical exergy of main fuel components.

Species	Mole fraction	Standard chemical exergy, $\bar{e}^{ch}_i$ (MJ/kmole)
CH_4	0.9227*	831.2
C_2H_6	0.0747*	1,495
C_3H_8	0.0026*	2,152.5

* Based on Table S6 in Supplemental Note 5.

The exergy of the engine exhaust flow is given as the sum of thermomechanical exergy, $\bar{e}_{th}$, and chemical exergy, $\bar{e}_{ch}$.

$$\dot{E}_{exh} = \dot{m}_{exh} \left(\bar{e}^{th}_{exh} + \bar{e}^{ch}_{exh}\right) \quad \text{(S3)}$$

The specific thermomechanical exergy of the engine exhaust flow is defined as

$$\bar{e}^{th}_{exh} = \bar{h}_{exh} - \bar{h}_0 - T_0(\bar{s}_{exh} - \bar{s}_0) \text{ and } \bar{s}_{exh} - \bar{s}_0 = \bar{s}^0_{exh} - \bar{s}^0_0 - \bar{R} \ln \frac{p_{exh}}{p_0}\,. \quad \text{(S4)}$$

Therefore, the specific thermomechanical exergy can be estimated using the following equation:

$$\bar{e}^{th}_{exh} = \bar{h}_{exh} - \bar{h}_0 - T_0 \left(\bar{s}^0_{exh} - \bar{s}^0_0 - \bar{R} \ln \frac{p_{exh}}{p_0}\right) \quad \text{(S5)}$$

where $\dot{m}_{exh}$ is the exhaust mole flow rate; $\bar{h}_{exh}$ and $\bar{s}_{exh}$ are the specific enthalpy and entropy of the engine exhaust at a state, respectively; and subscript $_0$ denotes the reference state at T_0 and p_0. In addition, $\bar{s}^0_{exh}$ is the absolute entropy of the exhaust flow, $\bar{R}$ is the universal gas constant, and p_{exh} is the pressure of the engine exhaust.

The chemical exergy of the engine exhaust flow can be estimated by

$$\bar{e}^{ch}_{exh} = \bar{R}T_0 \sum_{i=1}^{j} y_i \ln\left(\frac{y_i}{y_i^e}\right) = \sum_{i=1}^{j} y_i\, \bar{e}_i^{ch} + \bar{R}T_0 \sum_{i=1}^{j} y_i ln y_i \quad \text{(S6)}$$

where y_i and y_i^e are mole fractions of the ith species in the exhaust mixture at a state and in the reference environment, and $\bar{e}_i^{ch}$ is the standard chemical exergy, which can be obtained from the standard exergy table. The exhaust gas composition in the equation above was calculated by assuming the complete combustion. Thus, the global reaction equations of stoichiometric and 30% lean combustions employed are presented as follows:

At the stoichiometric modes:

$$0.9227CH_4 + 0.0747C_2H_6 + 0.0026C_3H_8 + 2.12(O_2 + 3.76N_2) \rightarrow 1.08CO_2 + 2.08H_2O + 7.97N_2 \quad \text{(S7)}$$

At the 30% lean modes:

$$0.9227CH_4 + 0.0747C_2H_6 + 0.0026C_3H_8 + 2.76(O_2 + 3.76N_2) \rightarrow 1.08CO_2 + 2.08H_2O + 0.64O_2 + 10.36N_2 \quad \text{(S8)}$$

The exergy of the coolant flow is given as

$$\dot{E}_{cool} = \dot{m}_{cool}Cp_{cool}\left((T_{cool} - T_0) - T_0 ln\left(\frac{T_{cool}}{T_0}\right)\right), \text{ and} \quad \text{(S9)}$$

the exergy of the engine heat loss is given as

$$\dot{E}_{eng,htls} = \dot{Q}_{eng,htls}\left(1 - \frac{T_0}{T_{eng}}\right), \quad \text{(S10)}$$

where $\dot{m}_{cool}$ and Cp_{cool} are the mass flow rate and specific heat of the engine coolant, respectively; T_{cool} is the coolant temperature; $\dot{Q}_{eng,htls}$ is the engine heat loss; T_{eng} is the engine surface temperature; and $\dot{Q}_{eng,htls}$ is estimated based on the remaining heat of fuel energy minus power, exhaust energy and coolant energy.

Thus, based on Eqs. (S1)–(S10), a thorough exergy analysis of the engine component was conducted to account for the exergy associated with fuel, work, exhaust gas, engine coolant, engine heat loss, and mechanical work. The exergy destroyed during the irreversible combustion process is determined by contrasting the exergy of the fuel with the residual exergy mentioned earlier in this section. In addition, the condensation of water in exhaust gas exiting the waste heat recovery system was considered based on the saturation pressure at the exhaust gas temperature exiting the waste heat recovery system.

For the waste heat recovery at the water tank, the exergy transfer from the exhaust gas and coolant to the water stored in the water tank was considered. The exergies available in the exhaust gas and coolant were used as inputs for the waste heat recovery component. To simplify the analysis, it was assumed that there was no additional heat loss between the engine and the waste heat recovery component. The exergy recovered from exhaust gas and coolant to the water tank was estimated based on the energy and exergy balances among exhaust gas, coolant, and tank water, shown in Eqs. (S11) and (S12).

$$\Delta Q_{exh} + \Delta Q_{cool} = \Delta Q_{water} + \Delta Q_{wt,loss} \quad \text{(S11)}$$

$$\Delta \dot{E}_{exh} + \Delta \dot{E}_{cool} = \dot{E}_{whr} + \dot{E}_{whr,loss} \quad \text{(S12)}$$

where ΔQ_{exh} is calculated based on the exhaust flow rate and temperature difference through the coil implemented in the water tank. A similar method is used to calculate ΔQ_{cool} based on coolant flow and their temperatures at the inlet and exit of the coil implemented in the water tank. Water tank heat loss, $\Delta Q_{wt,loss}$, is small owing to its excellent insulation (see Figure 1) and is assumed to be 0.1 kW. In addition, $\Delta \dot{E}_{exh}$ and $\Delta \dot{E}_{cool}$ are the exergy variations through the exhaust and coolant coils, respectively, in the water tank; $\dot{E}_{whr}$. and $\dot{E}_{whr,loss}$ are the recovered exergy and exergy loss in the water tank, respectively; and $\Delta \dot{E}_{exh}$, $\Delta \dot{E}_{cool}$ and $\dot{E}_{whr}$ are analyzed based on Eq. (S9) or Eq. (S10), respectively.

For electrical components such as a generator and a rectifier, most of the terms in Eq. (S1) can be ignored, except for the $\dot{W}$ and $\dot{E}_d$. Electricity generation was considered as $\dot{W}$. The loss at the electrical components was given as

$$\dot{W}_{in} - \dot{W}_{out} = \dot{E}_d. \quad \text{(S13)}$$

- **Discussion of the entire CHP system analysis**

In the CHP system analysis, fuel exergy shall be equal to the sum of electric generation, heat loss, exhaust loss, and destruction exergy at each component. In Eq. (S14), $\dot{E}_{d,ic\&hl}$ is exergy loss due to irreversible combustion and heat loss from the combustion chamber to coolant and oil.

$$\dot{E}_{fuel} = \dot{W}_{electric} + \sum \dot{E}_{heat} + \sum \dot{E}_{exh\ loss} + \sum \dot{E}_{d,ic\&hl} \quad \text{(S14)}$$

Table S12 summarizes the exergy flow and destruction in all 10 cases operating under the conditions listed in Supplemental Notes 2, 6, and 7. Figure S9 exhibits the Sankey diagrams of exergy flow and destruction in each component. Table S13 summarizes the energy flow and component loss in all 10 cases. Figure S10 further shows the Sankey diagrams of energy flow and losses in each component in all 10 cases.

Table S12. Summary of exergy flow and destruction of the 10 cases.

Case	Case 1	Case 2	Case 3	Case 4	Case 5	Case 6	Case 7	Case 8	Case 9	Case 10
Combustion mode	Stoich	Stoich	Stoich	Stoich	Stoich	Stoich	Lean	Lean	Lean	Lean
DC power (kW)	3.74	4.42	7.40	3.20	4.50	5.94	3.50	4.43	5.70	5.74
AC power (kW)	3.90	4.60	7.71	3.33	4.69	6.18	3.65	4.61	5.94	5.98
Water tank exergy recovered (kW)	0.81	0.87	2.37	0.71	1.176	0.74	0.87	0.72	0.77	0.67
Exergy electrical efficiency, DC (%)	15.4	16.5	24.2	15.4	16.8	23.5	20.9	27.3	32.4	32.0
Exergy electrical efficiency, AC (%)	16.1	17.2	25.2	16.0	17.5	24.5	21.8	28.5	33.8	33.4
Total exergy efficiency, DC (%)	18.8	19.8	32.0	18.8	21.2	26.5	26.0	31.8	36.8	35.8
Total exergy efficiency, AC (%)	19.4	20.4	33.0	19.5	21.9	27.5	26.9	32.9	38.1	37.1
Exhaust loss, destroyed (kW)	0.58	0.66	1.12	0.81	0.66	0.88	0.38	0.37	0.40	0.41
Engine heat loss, destroyed (kW)	0.18	0.20	0.24	0.16	0.20	0.22	0.13	0.13	0.17	0.15
Engine, destroyed (kW)	14.43	15.80	15.12	11.00	15.23	9.65	8.44	7.25	7.01	7.30
Generator, destroyed (kW)	0.43	0.51	0.856	0.37	0.52	0.69	0.41	0.51	0.66	0.66
Rectifier, destroyed (kW)	0.16	0.18	0.31	0.13	0.19	0.25	0.15	0.18	0.24	0.24
Water tank heat loss, destroyed (kW)	0.00	0.00	0.00	0.00	0.00	0.00	0.00	0.00	0.00	0.00
Water tank, destroyed (kW)	3.92	4.13	3.135	4.38	4.309	6.86	2.90	2.60	2.63	2.73
Total exergy loss (kW)	19.71	21.49	20.78	16.85	21.12	18.55	12.40	11.06	11.11	11.50
Generator exergy input (kW)	4.33	5.12	8.56	3.70	5.21	6.87	4.05	5.13	6.60	6.64
Rectifier exergy input (kW)	3.90	4.60	7.71	3.33	4.69	6.18	3.65	4.61	5.94	5.98
Engine-out exhaust exergy (kW)	4.90	5.57	5.82	5.48	5.57	7.96	3.63	3.21	3.39	3.50
Engine-out coolant exergy (kW)	0.41	0.09	0.80	0.42	0.58	0.54	0.52	0.48	0.41	0.32
Fuel exergy input (kW)	24.25	26.78	30.55	20.76	26.80	25.23	16.77	16.20	17.58	17.91

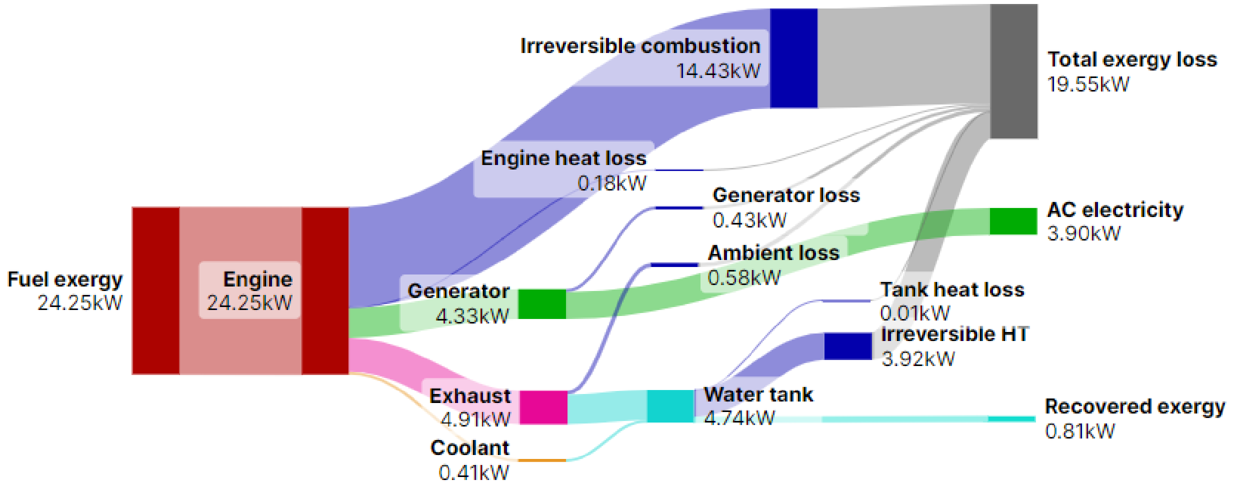

(a) Case 1

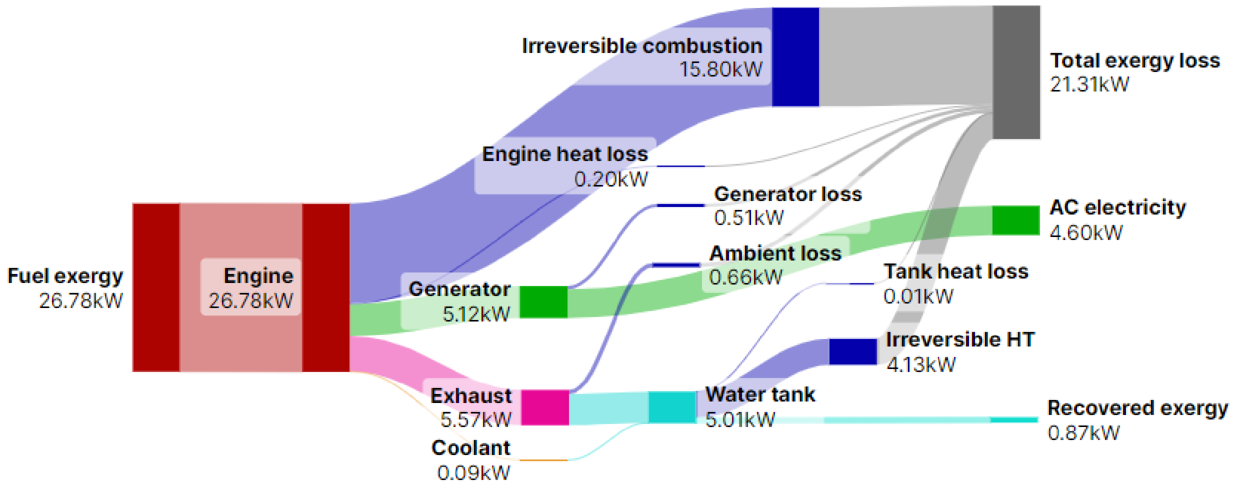

(b) Case 2

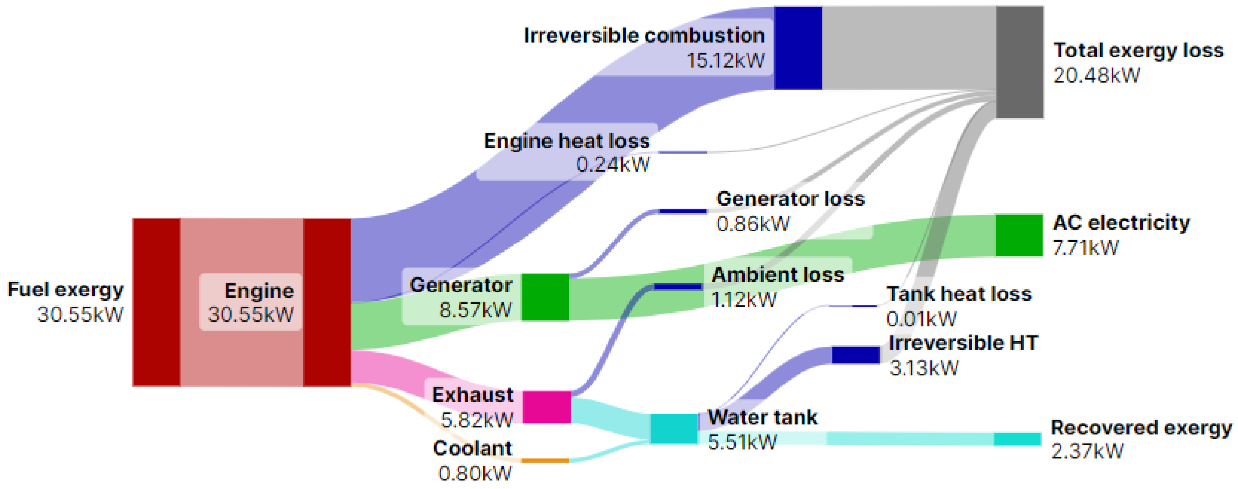

(c) Case 3

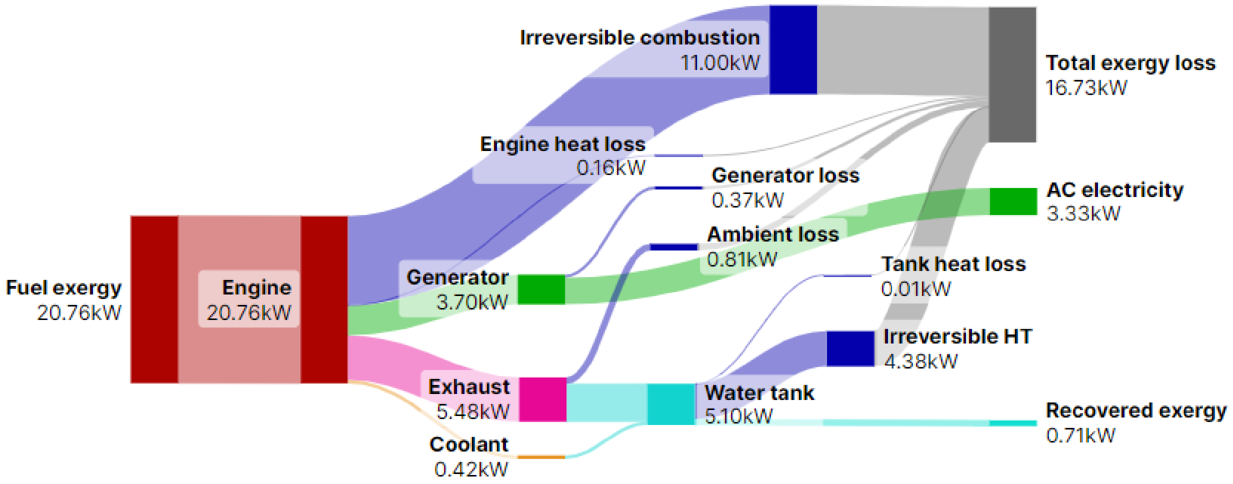

(d) Case 4

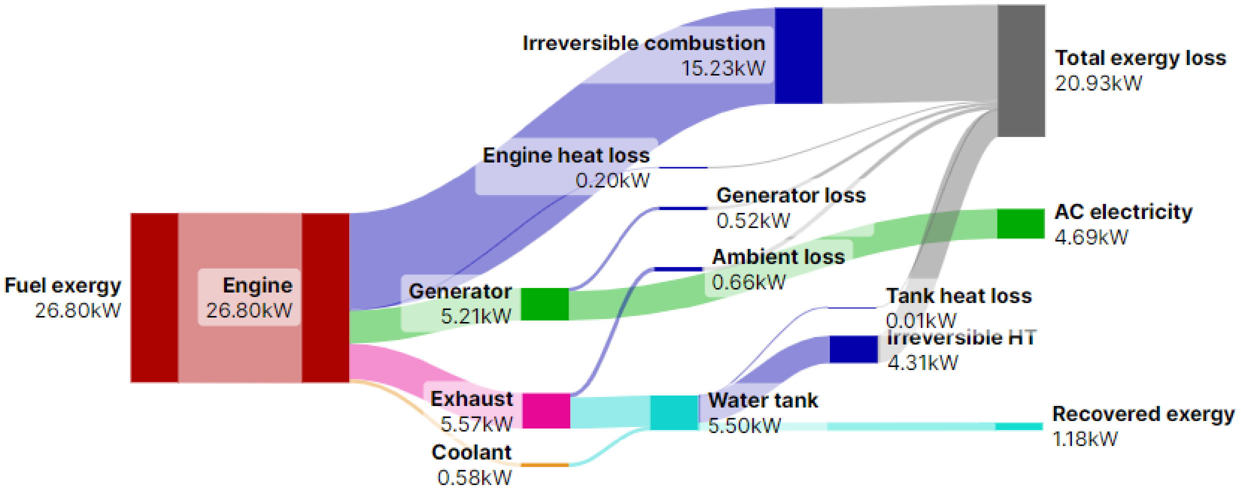

(e) Case 5

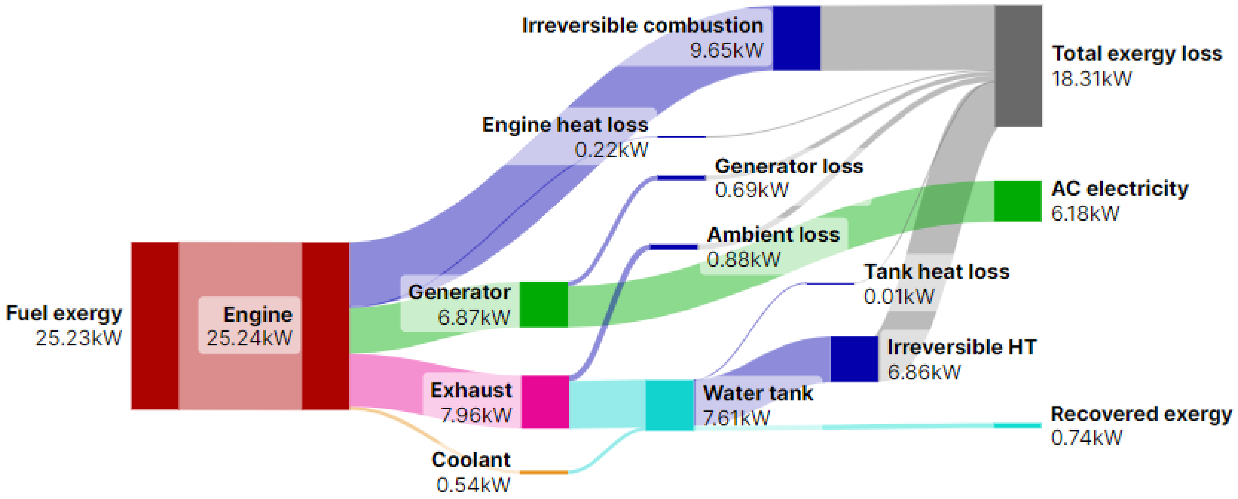

(f) Case 6

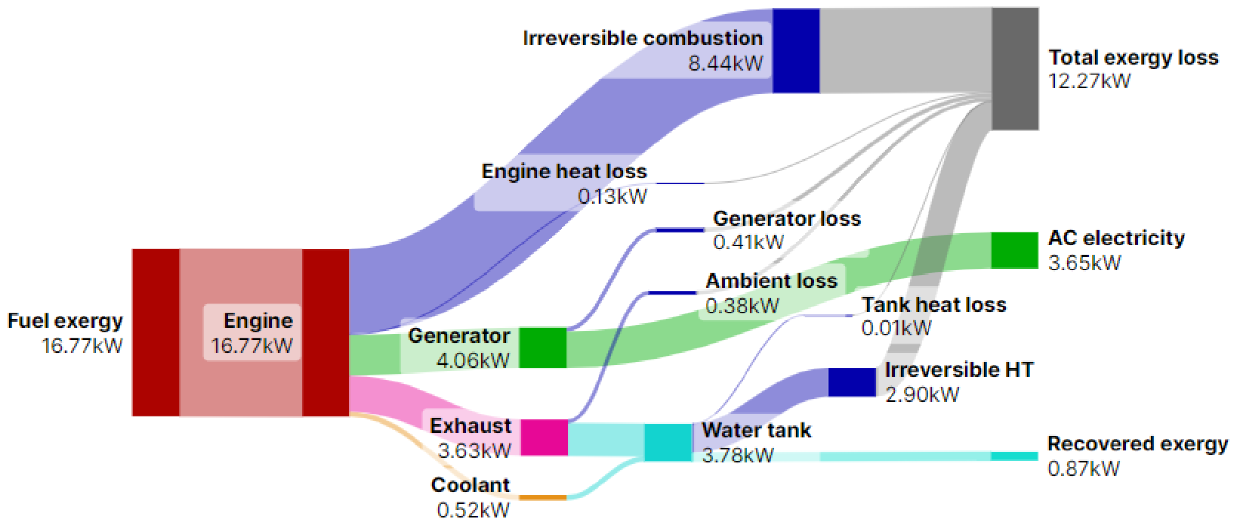

(g) Case 7

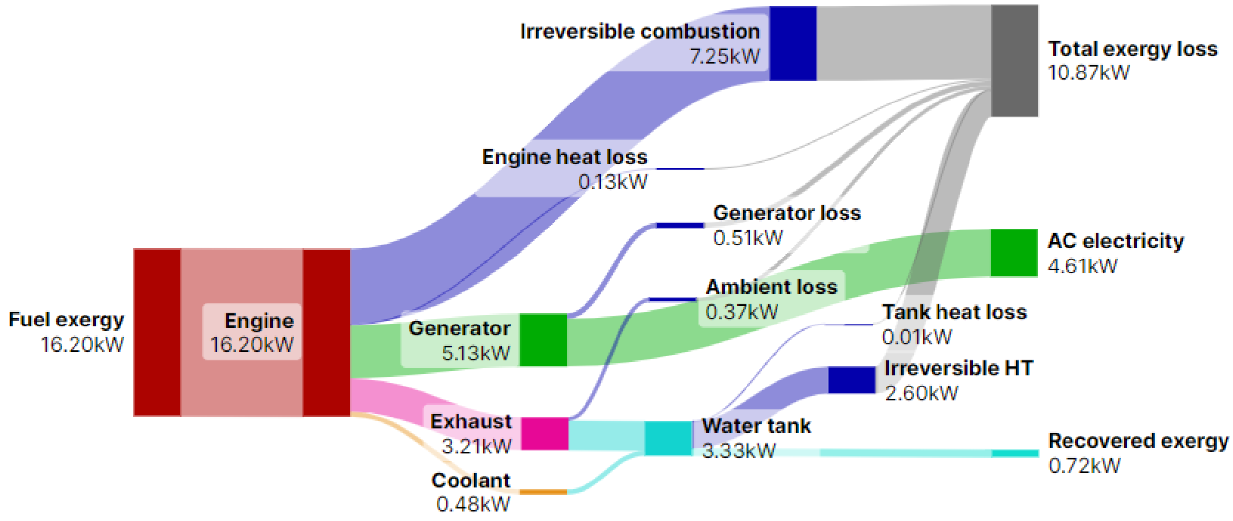

(h) Case 8

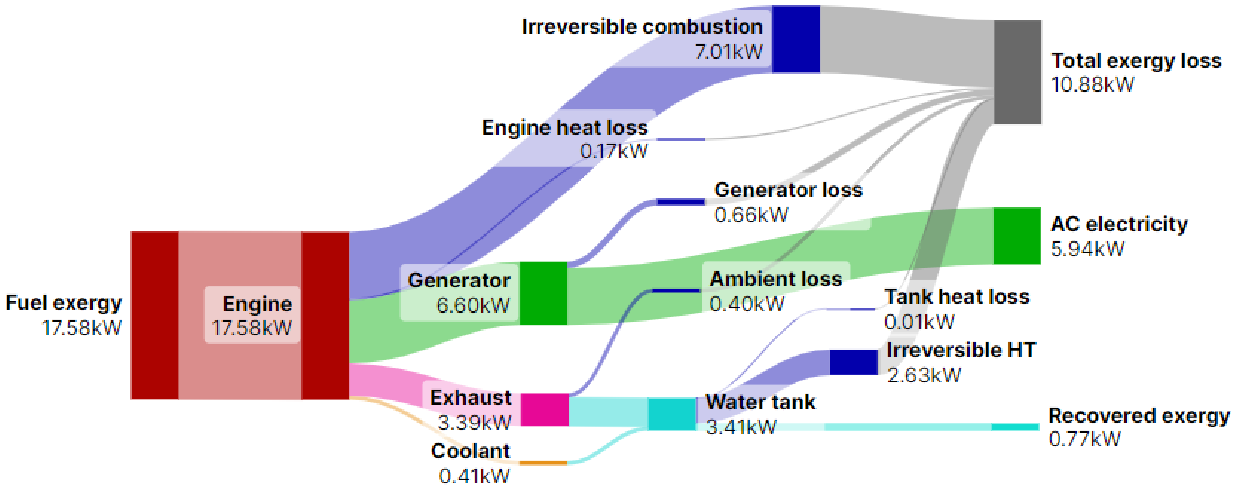

(i) Case 9

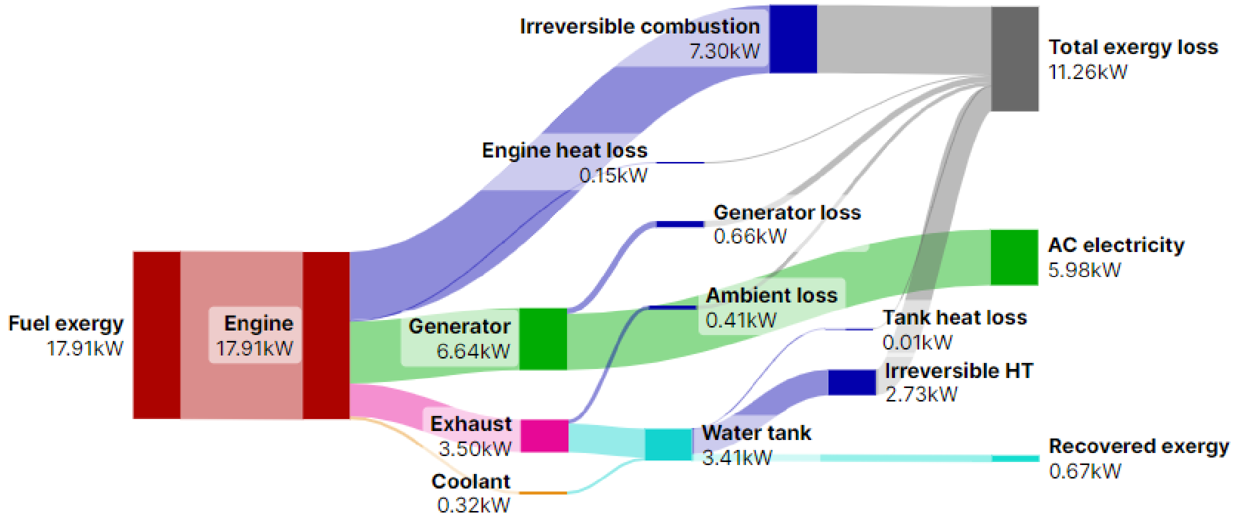

(j) Case 10

Figure S9. Exergy analysis of the mCHP system in the 10 cases operating under the conditions listed in Supplemental Notes 2, 6, and 7. Note: exergy loss for irreversible combustion includes exergy loss for heat loss from the combustion chamber to coolant and oil.

Table S13. Summary of energy flow and loss of the 10 cases.

Case	Case 1	Case 2	Case 3	Case 4	Case 5	Case 6	Case 7	Case 8	Case 9	Case 10
Combustion mode	Stoich	Stoich	Stoich	Stoich	Stoich	Stoich	Lean	Lean	Lean	Lean
DC power (kW)	3.74	4.418	7.39	3.194	4.546	5.935	3.497	4.432	5.697	5.74
AC power (kW)	3.90	4.60	7.70	3.33	4.74	6.18	3.64	4.62	5.93	5.98
Total energy efficiency, DC (%)	92.63	91.55	81.19	83.61	91.67	81.16	87.13	87.41	85.13	87.12
Total energy efficiency, AC (%)	93.23	92.19	82.13	84.21	92.34	82.08	87.96	88.47	86.40	88.37
Exhaust waste recovery (kW)	8.402	9.2	6.726	5.376	9.497	5.533	5.777	5.43	5.517	6.06
Coolant waste recovery (kW)	11.721	12.419	12.244	9.86	12.033	10.291	6.241	5.471	4.684	4.773
DC efficiency (%)	16.48	17.21	25.22	16.06	17.71	24.53	21.76	28.00	33.80	33.44
AC efficiency (%)	17.17	17.93	26.28	16.72	18.45	25.55	22.67	29.16	35.21	34.83
Engine efficiency (%)	19.07	19.92	29.19	18.58	20.50	28.39	25.19	32.40	39.13	38.70
Exhaust loss (kW)	0.523	0.922	4.307	2.402	0.843	3.439	1.299	1.175	1.465	1.151
Generator loss (kW)	0.433	0.511	0.855	0.37	0.526	0.687	0.405	0.513	0.659	0.664
Rectifier loss (kW)	0.156	0.184	0.308	0.133	0.189	0.247	0.146	0.185	0.237	0.239
Engine heat loss (kW)	0.688	0.686	0.538	0.609	0.71	0.577	0.342	0.236	0.314	0.295
Tank loss (kW)	0.1	0.1	0.1	0.1	0.1	0.1	0.1	0.1	0.1	0.1
Generator input (kW)	4.329	5.113	8.553	3.697	5.261	6.869	4.048	5.13	6.593	6.643
Rectifier input (kW)	3.896	4.602	7.698	3.327	4.735	6.182	3.643	4.617	5.934	5.979
Engine-out exhaust (kW)	9.025	10.222	11.133	7.878	10.44	9.072	7.176	6.705	7.082	7.311
Fuel input (HHV) (kW)	25.76	28.44	32.47	22.04	28.44	26.81	17.81	17.54	18.68	19.02
Fuel input (LHV) (kW)	23.27	25.68	29.32	19.91	25.69	24.21	16.08	15.84	16.87	17.18

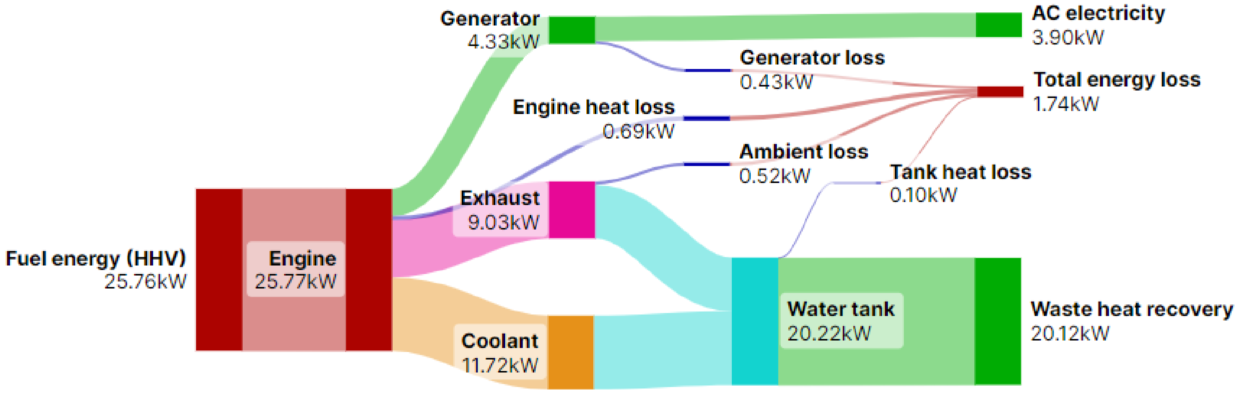

(a) Case 1

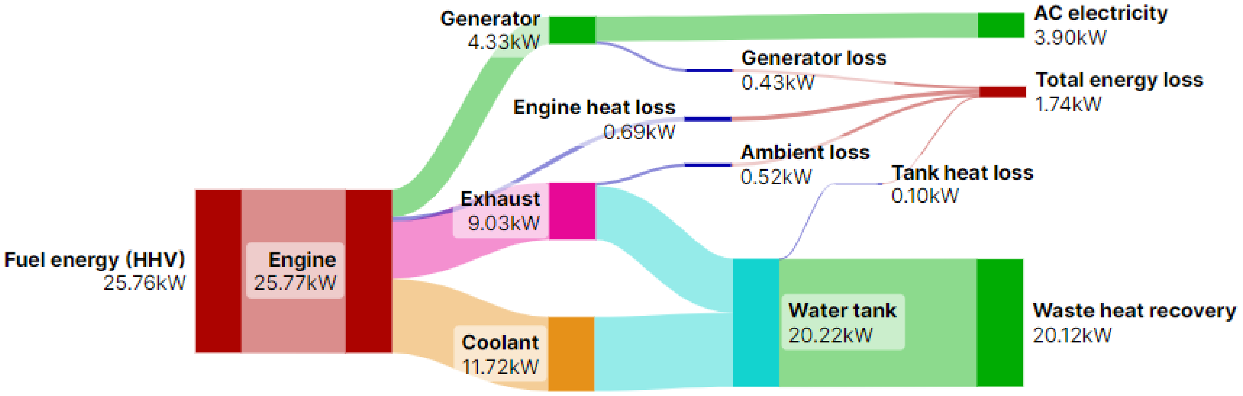

(b) Case 2

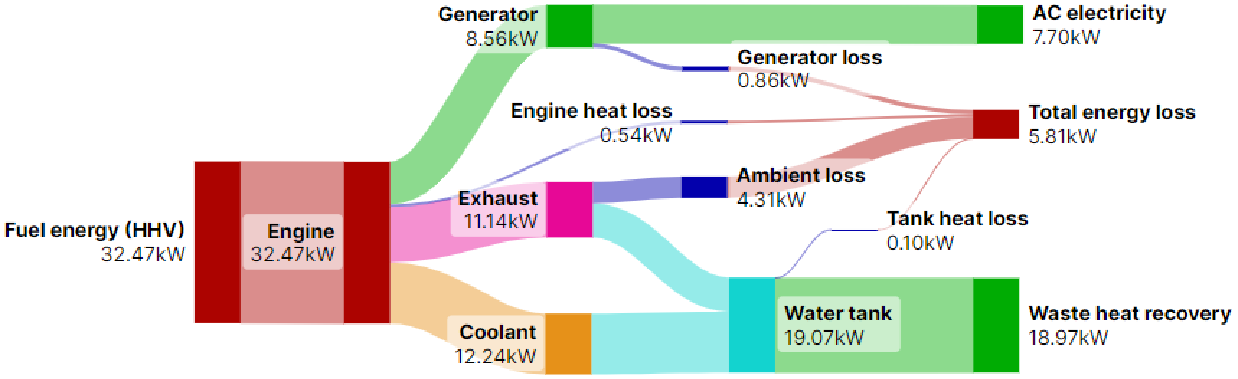

(c) Case 3

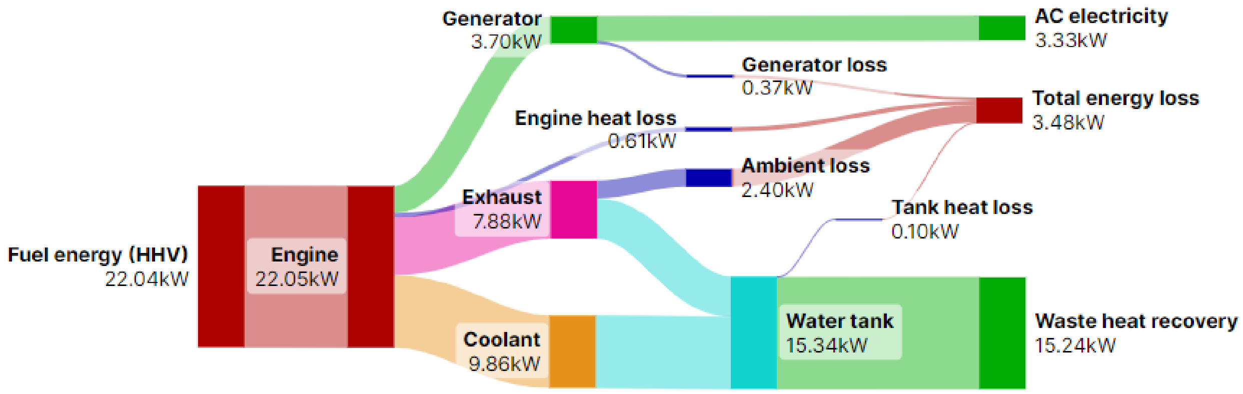

(d) Case 4

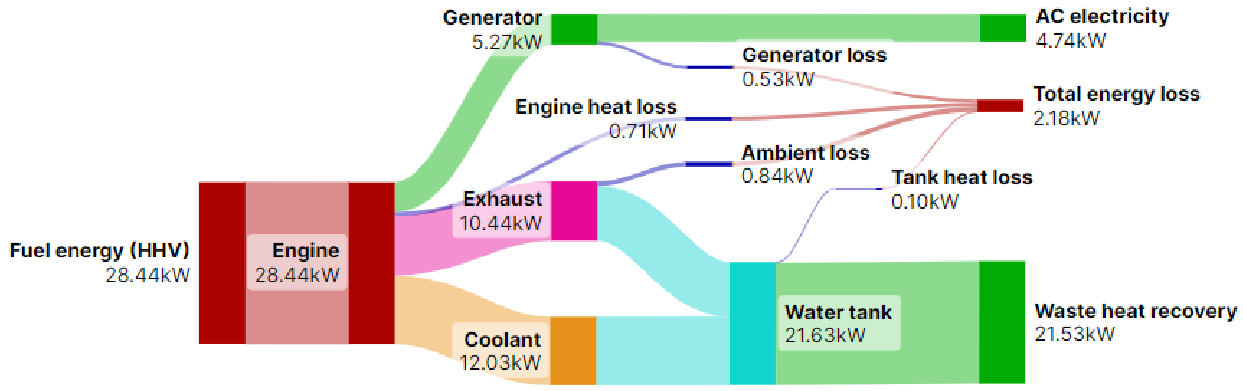

(e) Case 5

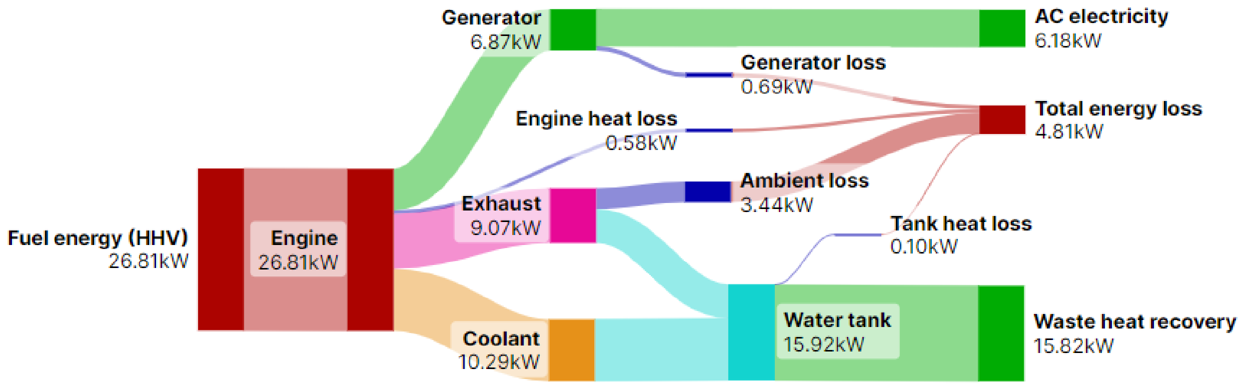

(f) Case 6

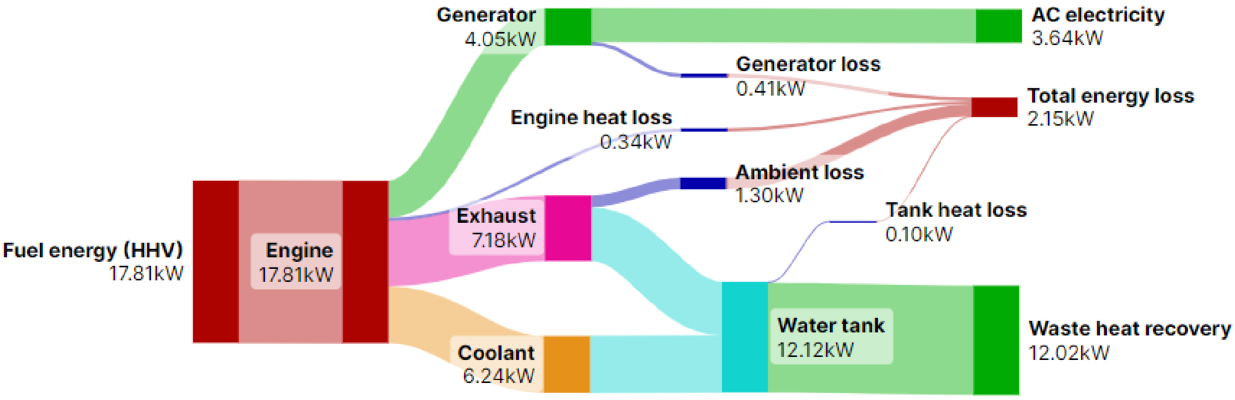

(g) Case 7

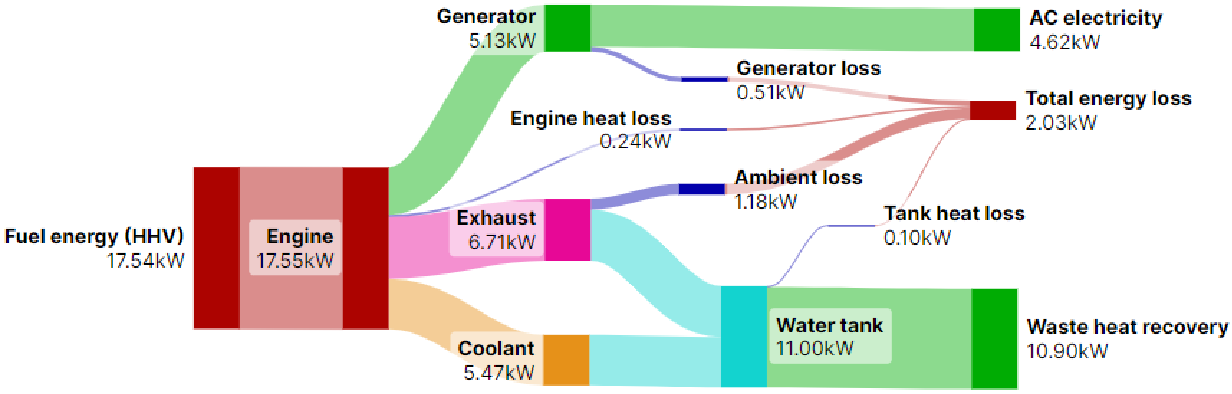

(h) Case 8

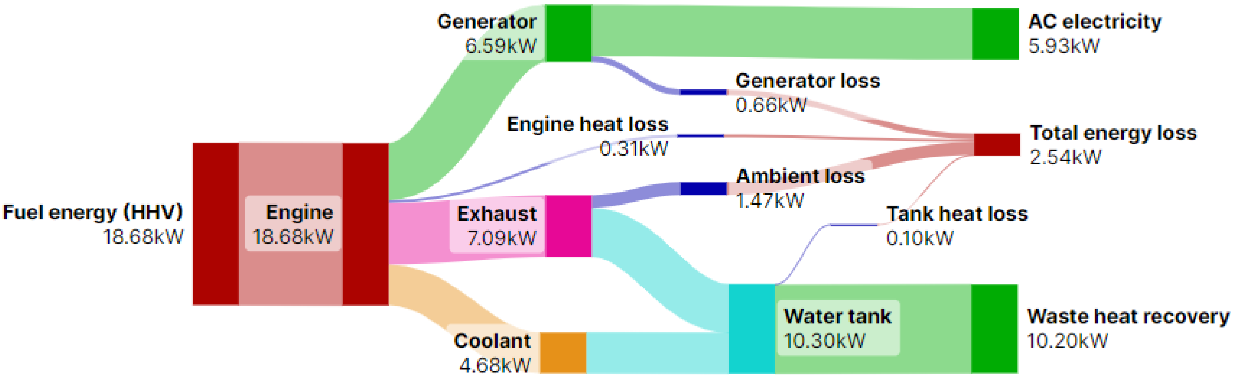

(i) Case 9

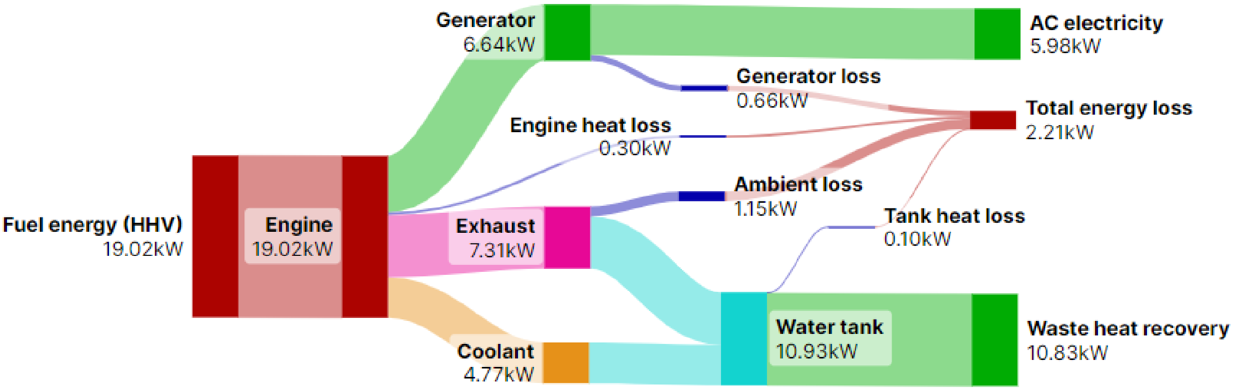

(j) Case 10

Figure S10. Energy analysis of the mCHP system in the 10 cases operating under the conditions listed in Supplemental Notes 2, 6, and 7.

Supplemental Note 16: Fuel types of the data shown in Figure 6

Table S14 summarizes fuel types of the data shown in Figure 6. The majority of fuels used in the data are natural gas. This is expected owing to natural gas's complete pipeline infrastructure and mature market.

Table S14. Fuel types of the data shown in Figure 6.

mCHP models available in market	Reference	Fuel type
OPS (ICE)	—	Natural gas
COGEN Microsystems (ORC)	[2]	Natural gas
Energetix: Genlec (ORC)	[2]	Natural gas
JX Crystal Prototype (TPV)	[2]	Natural gas
Senertec: Dachs (ICE)	[2, 4, 6, 8]	Natural gas
Solo: Solo 161 (Stirling)	[2,4, 6, 12]	Natural gas
Honda: Ecowill (ICE)	[2, 3]	Natural gas
Whisper Tech (Stirling)	[4, 13]	Natural gas
Academic prototype (ORC)	[14]	Biogas
Academic prototype (TPV)	[15]	Natural gas
Stirling Denmark: SM5A (Stirling)	[6]	Natural gas
EC POWER: XRGI® 6 (ICE)	[1]	Natural gas, propane, butane
EC POWER: XRGI®9 (ICE)	[1]	Natural gas, propane, butane
AEG: Ecopower (ICE)	[7]	Natural gas, propane
TOTEM: TOTEM 10 (ICE)	[9]	Methane
YANMAR: CP5WN (ICE)	[10]	Natural gas

Supplemental Note 17: Ten representative homes covering northern, middle, and southern climate zones

In the United States in 2023, electricity was produced from wide ranging sources such as coal (14.9%), hydroelectric (6.2%), natural gas (40.1%), nuclear power (19.6%), solar (4.4%), wind energy (13.5%), and others (2.3%) [22]. Fossil fuels such as coal and natural gas are still predominant, but renewable sources such as wind and solar are growing quickly in several states. For example, Texas led all US states in renewable energy production, accounting for over 23% of the nation's totals. Consequently, carbon intensity of electricity generation (i.e., kilograms of CO_2 per kilowatt-hour of power generation), as well as electricity retail price, vary substantially at different locations. To reasonably evaluate the benefits and disadvantages of the mCHP prototype performance in the decarbonization and operation cost savings of single household applications, 10 homes were selected, respectively, from cities in 10 states representing northern, middle, and southern climate zones in the United States. Figure S11 shows the locations of these representative homes as well as their different CO_2 kilograms per kilowatt-hour of power generation and diverse retail prices for electricity and natural gas. Figure S11 also shows the data for kilograms of CO_2 per kilowatt-hour of power generation, electricity retail prices, and natural gas retail prices, which are adopted from US Energy Information Administration data [23, 24, 25].

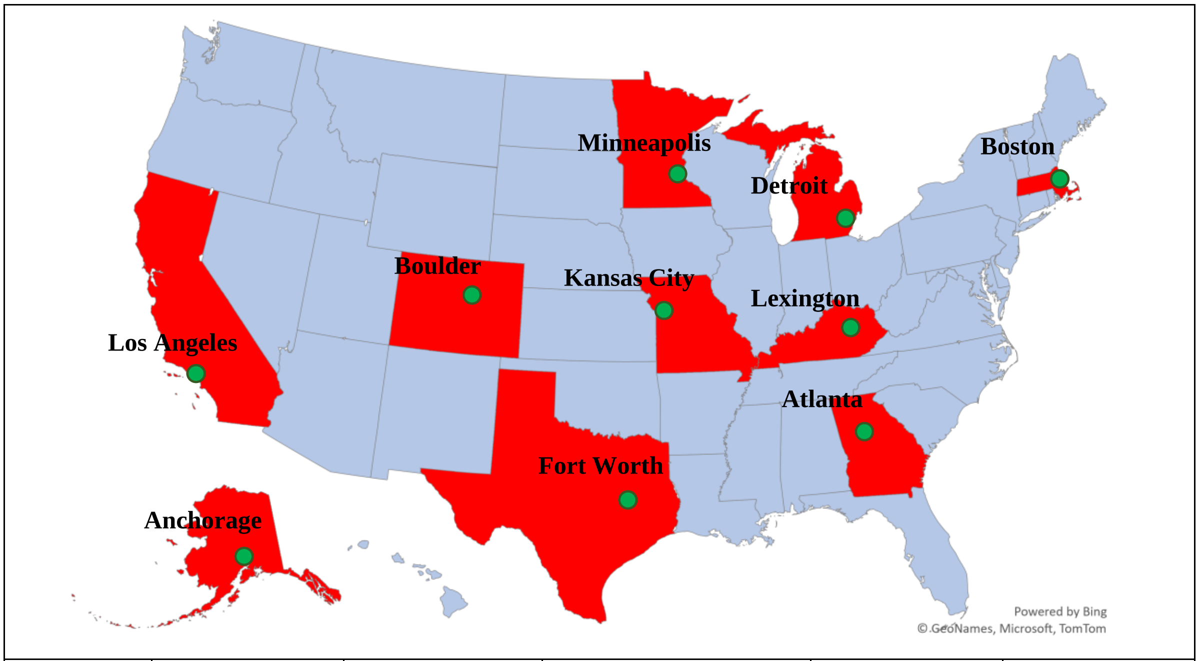

Zone	City	State	CO_2 per kilowatt-hour of power generation (g/kWh)	Electricity retail price (cents/kWh)	Natural gas retail price (cents/kWh)
Northern	Anchorage	Alaska	539.27	19.82	3.73
	Boston	Massachusetts	430.65	18.19	5.47
	Detroit	Michigan	476.52	12.21	3.14
	Minneapolis	Minnesota	391.52	10.57	3.21
Middle	Boulder	Colorado	547.66	10.27	3.11
	Kansas City	Missouri	775.31	9.64	3.69
	Lexington	Kentucky	803.28	8.58	4.17
Southern	Atlanta	Georgia	350.77	9.93	5.43
	Fort Worth	Texas	427.88	8.36	4.70
	Los Angeles	California	228.61	18.00	5.58

Figure S11. Ten representative homes covering northern, middle, and southern climate zones in the United States (map), indicating different carbon intensities of electricity generation and different retail prices for electricity and natural gas (table).

Figure S12 shows 1-year data of real-world electricity consumption and natural gas for space and water heating in the homes, downloaded from the EnergyPlus residential energy consumption database [26]. This database covers 1 year of real-world hour-by-hour measurements from 237 single houses across all 50 US states. Figure S13 compares the annual household electricity and heating gas consumption of the 10 households and clearly reflects that these homes experience different load demand because of diverse climate conditions. In the northern climate zone, the four homes in Anchorage, Boston, Detroit, and Minneapolis require substantial natural gas for space heating between November and April, and they still must use natural gas for water heating even between May and October. However, the homes in the southern climate zone require substantially

less natural gas for space heating between November and April, and no natural gas was used in Fort Worth. Additionally, the 10 homes require near-zero natural gas consumption in summertime (i.e., July to August). By contrast, annual electricity consumption of the homes in the southern climate zone is higher than that of the homes in the northern climate zone. The information is useful for effectively understanding the potential benefits and disadvantages of the mCHP prototype in US household applications.

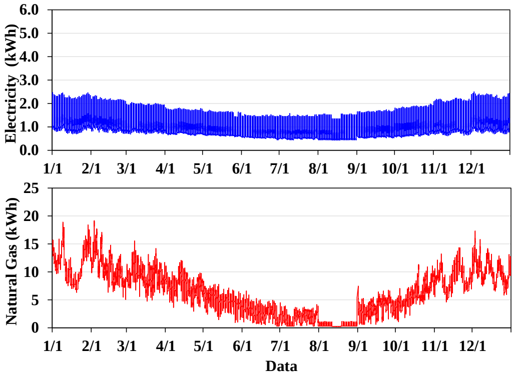

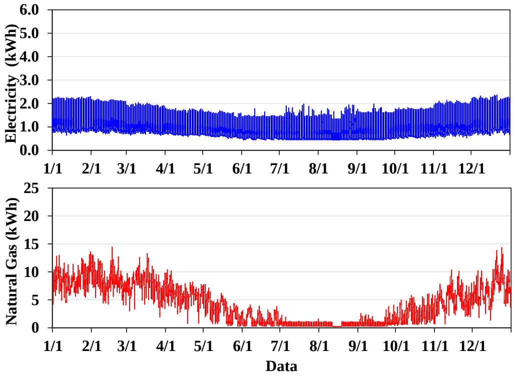

(a) Anchorage, Alaska (b) Boston, Massachusetts

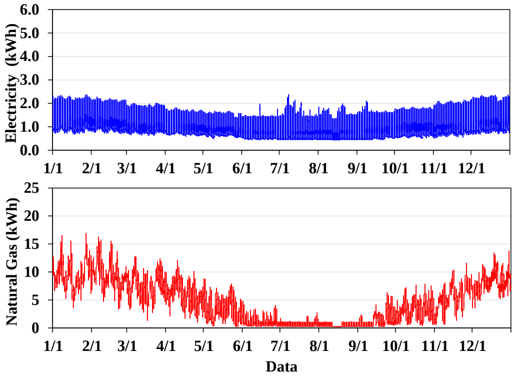

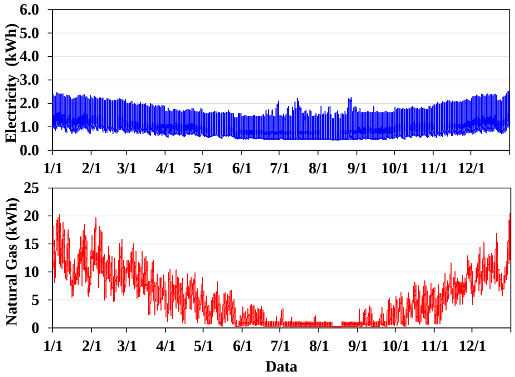

(c) Detroit, Michigan (d) Minneapolis, Minnesota

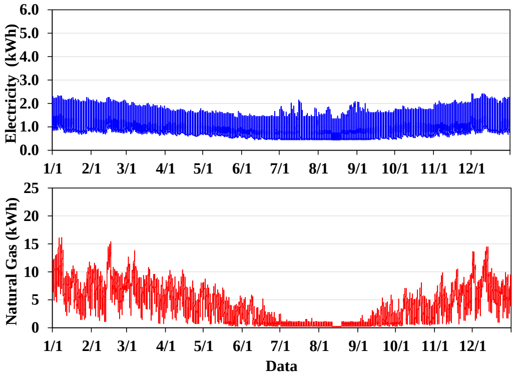

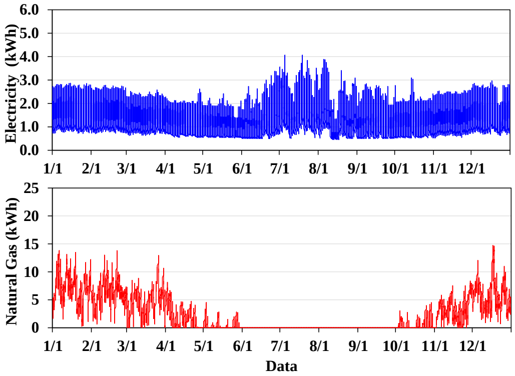

(e) Boulder, Colorado (f) Kansas City, Missouri

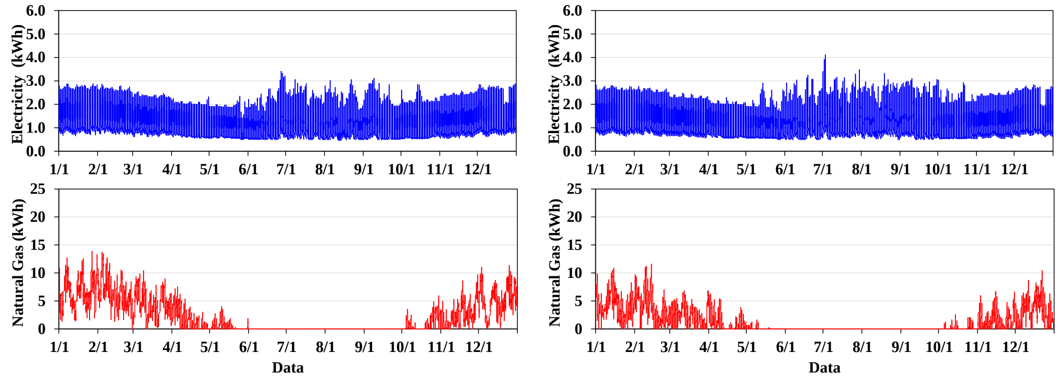

(g) Lexington, Kentucky (h) Atlanta, Georgia

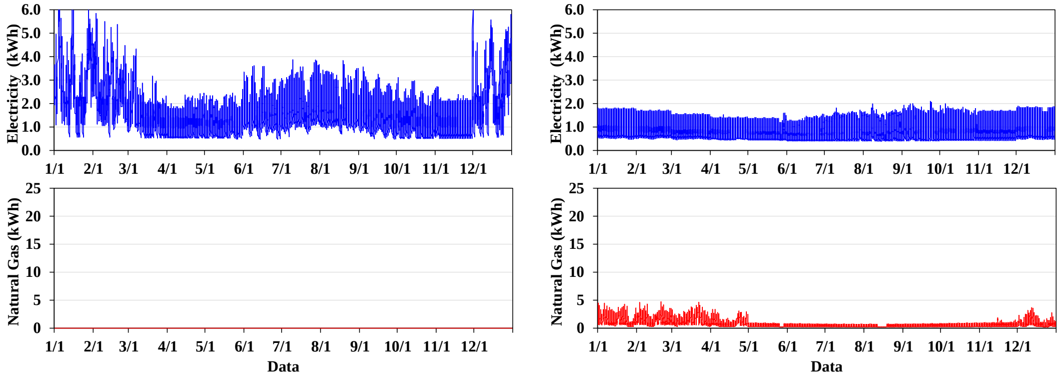

(i) Fort Worth, Texas (j) Los Angeles, California

Figure S12. One year's data of real-world electricity and natural gas consumption for space and water heating of the 10 representative homes located in Anchorage, Alaska; Boston, Massachusetts; Detroit, Michigan; Minneapolis, Minnesota; Boulder, Colorado; Kansas City, Missouri; Lexington, Kentucky; Atlanta, Georgia; Fort Worth, Texas; and Los Angeles, California.

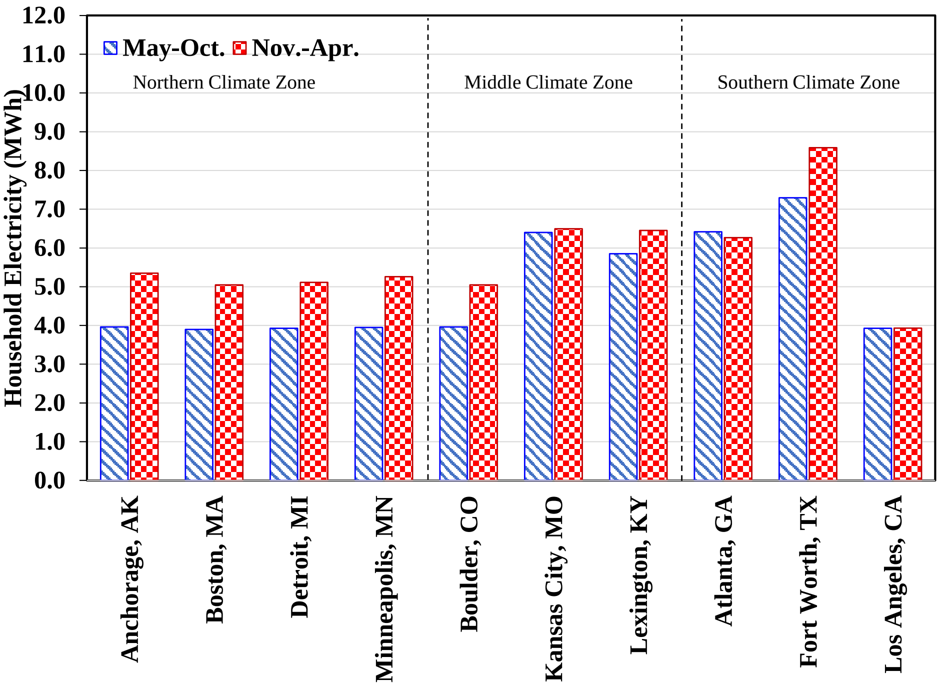

(a) Annual household electricity

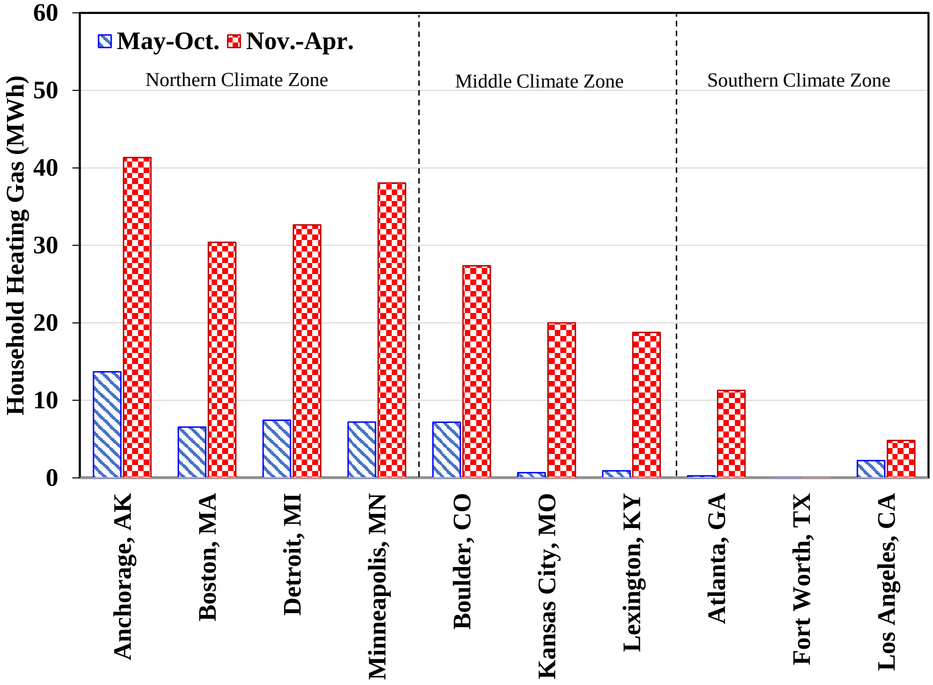

(b) Annual heating gas consumption

Figure S13. Annual household electricity and heating gas consumption of the 10 households.

Supplemental Note 18: Life cycle analysis

The life cycle analysis for the mCHP was assessed over 20 years. In the analysis, the mCHP is assumed to replace residential furnace, water heater, and grid power supply, which are typically equipped in a representative home. The life span of a residential furnace and water heater adopts a typical time of 10 years. Also, the battery system used in the mCHP is assumed to have a life span of 10 years. Thus, the residential furnace and water heater, as well as the battery system, shall be replaced at the end of year 10. The detailed life cycle analysis of mCHP accounts for annual operation cost savings, annual maintenance cost savings, initial investment penalty and replacement cost savings, and disposal cost savings at the end of the device life span (including initial investment and device replacement). Thus, the net present value savings (ΔC_{NPV}) is used to evaluate the economic feasibility of the mCHP.

$$\Delta C_{NPV} = \sum_{i=1}^{i=n_{mchp}} \frac{(\Delta C_{AOS}(i)+\Delta C_{MAINT}(i))(1+\alpha)^{i-1}}{(1+\beta)^{i}} + \Delta C_{INV} + \Delta C_{REP} + \Delta C_{DSP} \quad \text{(S15)}$$

where $\Delta C_{AOS}(i)$ and $\Delta C_{MAINT}(i)$ are annual operation cost savings and annual maintenance cost savings in the ith year, respectively; ΔC_{INV} is the initial investment savings of the mCHP purchase and installation; ΔC_{REP} is the replacement cost savings for residential furnace and water heater compared with replacing the mCHP battery system after 10 years; ΔC_{DSP} is the disposal cost savings of the mCHP compared with residential furnace and water heater; and α and β are the inflation rate and the discount rate, respectively. Also, $n_{mchp} = 20$ is the life span of mCHP. Next, $\Delta C_{AOS}(i)$, $\Delta C_{MAINT}(i)$, ΔC_{INV}, ΔC_{REP}, and ΔC_{DSP} are evaluated by using the following equations:

$$\Delta C_{AOS}(i) = \sum_{k=0}^{k=1yr} \left(\dot{E}_{h\_ng}(k) \cdot \alpha_{ng} + \dot{W}_{h\_ee}(k) \cdot \alpha_{ee} - \dot{E}_{mchp_{ng}}(k) \cdot \alpha_{ng} - \dot{W}_{mchp\_ee}(k) \cdot \alpha_{ee} \right) \quad \text{(S16)}$$

$$\Delta C_{MAINT}(i) = c_{fn,maint} + c_{wh,maint} - c_{mchp,maint} \quad \text{(S17)}$$

$$\Delta C_{INV} = \left(c_{fn,dev} + c_{fn,ins} + c_{wh,dev} + c_{wh,ins}\right) - \left(c_{mchp,dev} + c_{mchp,ins} + c_{batt,dev} + c_{batt,ins}\right) \quad \text{(S18)}$$

$$\Delta C_{REP} = \left(c_{fn,dev}(1+\alpha)^{n_{fn}-1} + c_{wh,dev}(1+\alpha)^{n_{wh}-1}\right) - c_{batt,dev}(1+\alpha)^{n_{batt}-1} \quad \text{(S19)}$$

$$\Delta C_{DSP} = C_{DSP,fn} + C_{DSP,wh} - C_{DSP,mchp} - C_{DSP,batt} \quad \text{(S20)}$$

and

$$C_{DSP,fn} = c_{fn,dsp}(1+\alpha)^{n_{fn}-1}(1+(1+\alpha)^{n_{fn}}) \quad \text{(S21)}$$

$$C_{DSP,wh} = c_{wh,dsp}(1+\alpha)^{n_{wh}-1}(1+(1+\alpha)^{n_{wh}}) \quad \text{(S22)}$$

$$C_{DSP,mchp} = c_{mchp,dsp}(1+\alpha)^{n_{mchp}-1} \quad \text{(S23)}$$

$$C_{DSP,batt} = c_{batt,dsp}(1+\alpha)^{n_{batt}-1}(1+(1+\alpha)^{n_{batt}}) \quad \text{(S24)}$$

where $\dot{E}_{h\_ng}(k)$ is home thermal energy demand using natural gas; $\dot{W}_{h\_ee}(k)$ is home grid power demand; $\dot{E}_{mchp_{ng}}(k)$ is mCHP natural gas consumption; $\dot{W}_{mchp\_ee}(k)$ is grid electricity buffering the excess power demand in mCHP applications; α_{ng} is residential natural gas price; α_{ee} is

residential electricity price; $c_{furn,maint}$, $c_{wh,maint}$, and $c_{mchp,maint}$ are the present maintenance cost for furnace, water heater, and mCHP, respectively; $c_{fn,dev}$, $c_{wh,dev}$, $c_{fn,ins}$, and $c_{wh,ins}$ are the present initial costs of the furnace and water heater, as well as their installation costs; $c_{mchp,dev}$, $c_{batt,dev}$, $c_{mchp,ins}$, and $c_{batt,ins}$ are the present initial costs of mCHP and battery systems, as well as their installation costs; $c_{fn,dsp}$, $c_{wh,dsp}$, $c_{mchp,dsp}$, and $c_{batt,dsp}$ are the current disposal costs for the furnace, water heater, mCHP, and battery; $C_{DSP,fn}$, $C_{DSP,wh}$, $C_{DSP,mchp}$, and $C_{DSP,batt}$ are the disposal costs of a furnace, water heater, mCHP, and battery at their life span (including their replacement disposal); and n_{fn}, n_{wh}, and n_{batt} are the life span of a residential furnace, water heater, and battery system. All the present initial, maintenance, and disposal costs of these device is listed in Table S15.

Table S15: Present initial, maintenance, and disposal costs of mCHP, battery, furnace and water heater.

**Component**	**Value ($)**	**Installation ($)**	**Annual repair/ maintenance ($)**	**Disposal ($)**	**Life span (years)**
mCHP[a]	4,400	3,600	550 (see Table S16)	300	20
mCHP accessory: 6 kWh battery[b]	2,500[i]			50	10
Residential 80 kBtu furnace[c,d,e]	1,460	3,240	325	280	10
50 gal natural gas water heater[f,g]	729	1,878	188	105	10
50 gal electrical heater[g,h]	529	648	188	105	10

[a] https://iea-etsap.org/E-TechDS/PDF/E04-CHP-GS-gct_ADfinal.pdf
[b] https://glginsights.com/articles/the-economics-around-lithium-ion-battery-recycling-are-strong-and-growing/
[c] https://www.theacoutlet.com/gm9s920803bn-goodman-80-000-btu-92-afue-9-speed-gas-furnace.html?gad_source=1&gclid=Cj0KCQiA3uGqBhDdARIsAFeJ5r1pWQK7RcNlxcPC_6Wzy6kMY4yMrqrzk2KW3g4ZfiCxcKmBS5tQ3IMaAu22EALw_wcB
[d] https://www.forbes.com/home-improvement/hvac/new-furnace-cost/#:~:text=The%20average%20cost%20of%20installing,the%20final%20cost%20for%20homeowners.
[e] https://myairauthority.com/furnace-tuneup-cost-breakdown/#:~:text=Annual%20maintenance%20can%20be%20between,%2C%20field%20type%2C%20and%20model.
[f] https://www.lowes.com/pd/A-O-Smith-Signature-50-Gallon-Tall-6-Year-Limited-Natural-Gas-Water-Heater/1000542577?cm_mmc=shp-_-c-_-prd-_-plb-_-ggl-_-LIA_PLB_209_Water-Heaters-_-1000542577-_-local-_-0-_-0&gad_source=4&gclid=Cj0KCQiA3uGqBhDdARIsAFeJ5r2eTZi9cUu3n_IMY70FD2YmkMCLxj37UfTI7TfRdQea-5zPWmXZMMgaAoOREALw_wcB&gclsrc=aw.ds
[g] https://todayshomeowner.com/plumbing/cost/water-heater-installation-cost/
[h] https://www.lowes.com/pd/A-O-Smith-Signature-50-Gallon-Short-6-year-Limited-4500-Watt-Double-Element-Electric-Water-Heater/1000216833?cm_mmc=shp-_-c-_-prd-_-plb-_-ggl-_-LIA_PLB_209_Water-Heaters-_-1000216833-_-local-_-0-_-0&gad_source=4&gclid=Cj0KCQiA3uGqBhDdARIsAFeJ5r1oDwSkeEOGKGFJ-6D4nyYf4uEBIcSMDfueNzbuwzlkmx5-Qthb4cQaAuRoEALw_wcB&gclsrc=aw.ds
[i] https://www.18650batterystore.com/products/bigbattery-grila-48v-120ah-6kwh-lifepo4

Table S16. Detailed mCHP annual repair and maintenance costs

**Annual repair and maintenance**	**Component cost and labor cost**
Oil and oil filter	$160
Spark plug	$220
Air filter	$120
Water pump	$50

In Eq. S16, $\dot{E}_{h\_ng}(k)$ and $\dot{W}_{h\_ee}(ki)$ are assessed based on the selected home data, and $\dot{E}_{mchp_{ng}}(k)$ and $\dot{W}_{mchp\_ee}(k)$ are determined by an operating strategy designed to maximally utilize the electricity and waste heat from the mCHP for a single household application. In that

strategy, the mCHP operates optimally by switching between stoichiometric and lean modes, considering a trade-off of cost savings and carbon emissions reduction while meeting thermal energy and electricity demands in a home. In each mCHP operation mode, the electricity output is used to satisfy household power demand. However, if the mCHP electricity output falls short of meeting household power demand, grid electricity buffers the excess power demand. Similarly, the mCHP waste heat is used to satisfy household thermal energy demand for space and water heating typically fulfilled by natural gas. However, if the mCHP waste heat is insufficient to satisfy thermal energy demand, the mCHP uses its electricity output, along with grid electricity, to buffer the additional thermal energy demand. This operating strategy aims to maximize the utilization of the mCHP energy output.

In the detailed strategy, the mCHP is assumed to run lean mode and stoichiometric modes (i.e., cases 5 and 9, respectively). Case 5 in the stoichiometric mode enables 4.74 AC kW (i.e., 4.55 DC kW) power output and generates 21.52 kW waste heat at 92.3% overall mCHP efficiency (based on the higher heating value) and 18.4% AC efficiency (based on the lower heating value). Case 9 in the stoichiometric mode enables 5.93 AC kW (i.e., 5.7 kW) power output and generates 11.77 kW waste heat at 86.4% overall mCHP efficiency (based on the higher heating value) and 35.2% AC efficiency (based on the lower heating value). Evidently, the stoichiometric mode provides lower power and high thermal energy, and the lean mode delivers high power and lower thermal energy for house usage. Based on the power and thermal energy demand from a given house, the mCHP adopts a targeted function (see Eq. [S25]) to optimally run either the lean or stoichiometric mode through minimizing the combined value of operation cost and carbon emissions. Tables S17 and S18 show the strategy of using the electricity and waste heat from the mCHP for a single household application.

$$\text{Obj} = \min\left(0.5\left(\frac{Cost_{chp,stoich}(k)}{Cost_{home}(k)} + \frac{CO2_{chp,stoich}(k)}{CO2_{home}(k)}\right), 0.5 * \left(\frac{Cost_{chp,lean}(k)}{Cost_{home}(k)} + \frac{CO2_{chp,lean}(k)}{CO2_{home}(k)}\right)\right) \quad \text{(S25)}$$

In addition, to evaluate the environmental effect of mCHP, life cycle CO_2 emission analysis was also performed based on mCHP's carbon emissions associated with gas consumption plus carbon emissions associated with electricity imported from the grid, minus CO_2 generated in gas and electricity consumption in residential home devices, given as below:

$$\Delta CO2_{1y} = \sum_{k=0}^{k=1yr}\left(\frac{\dot{E}_{h\_ng}(k)}{(1-\xi_{ng})} \cdot f_{CO2e_{ng}} + \frac{\dot{W}_{h_{ee}}(k)}{(1-\xi_{ee})} \cdot f_{CO2e_{ee}} - \frac{\dot{E}_{mchp_{ng}}(k)}{(1-\xi_{ng})} \cdot f_{CO2e_{ng}} - \frac{\dot{W}_{mchp\_ee}(k)}{(1-\xi_{ee})} \cdot f_{CO2e_{ee}}\right) \text{(S26)}$$

$$\Delta CO2_{lca} = n_{mchp} \cdot \Delta CO2_{1y} \quad \text{(S27)}$$

where $\Delta CO2_{1y}$ and $\Delta CO2_{lca}$ are CO_2 emissions reduction over the period of 1 year and mCHP life cycle. The equation above assumes US grid transmission and distribution loss ξ_{ee}= 4% [27]; US natural gas transmission and distribution loss ξ_{ng} = 5% [28]; residential natural gas price α_{ng} depends on the simulated locations [26], shown in Figure S11; residential electricity price α_{ee} depends on the simulated locations [25], shown in Figure S11; CO_2e per kilowatt electricity generation, $f_{CO2e_{ee}}$, depends on the simulated locations [24], also shown in Figure S9; and CO_2e per kilowatt natural gas consumption $f_{CO2e_{ng}} = 180.5$ g/kWh [29].

Table S17. Analysis of annual operation cost and CO_2 for a conventional home and a home using mCHP.

Input: lean-mode mCHP power output $\dot{W}_{chp,lean}$
lean-mode mCHP waste heat $\dot{E}_{chp_{whr},lean}$
lean-mode overall mCHP efficiency $\eta_{chp,lean_{tot}}$
stoich-mode mCHP power output $\dot{W}_{chp,stoich}$
stoich-mode mCHP waste heat $\dot{E}_{chp_{whr},stoich}$
stoich-mode overall mCHP efficiency $\eta_{chp_{tot},stoich}$
Home grid power demand $\dot{W}_{h\_ee}$
Home thermal energy demand using natural gas $\dot{E}_{h\_ng}$
Home furnace efficiency $\eta_{furnace}$
US grid transmission and distribution loss ξ_{ee}
US natural gas transmission and distribution loss ξ_{ng}
Residential electricity price α_{ee}
Residential natural gas price α_{ng}
CO_2e per kW electricity generation $f_{CO2e_{ee}}$
CO_2e per kW natural gas consumption $f_{CO2e_{ng}}$

Outputs: 1-year operation cost and CO_2 emissions for conventional home and home using mCHP

For time = 0 **to** 1 year

// $OC_{home}(k)$ and $CO2_{home}(k)$: the operation cost and CO_2 emissions of a home at a given time i
// $OC_{chp,lean}(k)$ and $CO2_{chp,lean}(k)$: the operation cost and CO_2 emissions of the mCHP at lean mode
//$OC_{chp,stoich}(k)$ and $CO2_{chp,stoich}(k)$: the operation cost and CO_2 emissions of the mCHP at stoichiometric mode

$Cost_{home}(k) = \dot{E}_{h\_ng} \cdot \alpha_{ng} + \dot{W}_{h\_ee} \cdot \alpha_{ee}$`

$CO2_{home}(k) = \dot{E}_{h\_ng} / \left((1 - \xi_{ng}) \cdot \eta_{furnace} \right) \cdot f_{CO2e_{ng}} + \dot{W}_{h\_ee} / (1 - \xi_{ee}) \cdot f_{CO2e_{ee}}$

Sub_mode (stoich mode**)**->mCHP command ε, grid demand $\dot{W}_{grid}$, waste heat loss $\dot{E}_{waste}$ @stoich mode

$Cost_{chp,stoich}(k) = \varepsilon \cdot \left(\dot{E}_{chp_{whr},stoich} + W_{chp,stoich} \right) / \eta_{chp_{tot},stoich} \cdot \alpha_{ng} + \dot{W}_{grid} \cdot \alpha_{ee}$

$CO2_{chp,stoich}(k) = \varepsilon \cdot \left(\dot{E}_{chp_{whr},stoich} + W_{chp,stoich} \right) / \left((1 - \xi_{ng}) \cdot \eta_{chp_{tot},stoich} \right) \cdot f_{CO2e_{ng}} + \dot{W}_{grid} / (1 - \xi_{ee}) \cdot f_{CO2e_{ee}}$

Sub_mode (Lean mode**)**->mCHP command ε, grid demand $\dot{W}_{grid}$, waste heat loss $\dot{E}_{waste}$

$Cost_{chp,lean}(k) = \varepsilon \cdot \left(\dot{E}_{chp_{whr},lean} + \dot{W}_{chp,lean} \right) / \eta_{chp_{tot},lean} \cdot \alpha_{ng} + \dot{W}_{grid} \cdot \alpha_{ee}$

$CO2_{chp,lean}(k) = \varepsilon \cdot \left(\dot{E}_{chp_{whr},lean} + \dot{W}_{chp,lean} \right) / \left((1 - \xi_{ng}) \cdot \eta_{\eta_{chp_{tot},lean}} \right) \cdot f_{CO2e_{ng}} + \dot{W}_{grid} / (1 - \xi_{ee}) \cdot f_{CO2e_{ee}}$

Obj=min(0.5*$\left(\frac{Cost_{chp,stoich}(i)}{Cost_{home}(i)} + \frac{CO2_{chp,stoich}(i)}{CO2_{home}(i)} \right), 0.5 * \left(\frac{Cost_{chp,lean}(i)}{Cost_{home}(i)} + \frac{CO2_{chp,lean}(i)}{CO2_{home}(i)} \right)$)

If (Obj==0.5 $* \left(Cost_{chp,lean}(k) + CO2_{chp,lean}(k) \right)$)
Run lean mode
$Cost_{chp}(k)$= $Cost_{chp,lean}$
$CO2_{chp}(k)$=$CO2_{chp,lean}$
Else
Run stoich mode
$Cost_{chp}(k)$= $Cost_{chp,stoich}$
$CO2_{chp}(k)$=$CO2_{chp,stoich}$
End
End

$1yCost_{home} = \sum_{1yr} Cost_{home}(k)$; $1yCO2_{home} = \sum_{1yr} CO2_{home}(k)$;
$1yCost_{chp} = \sum_{1yr} Cost_{chp}(k)$; $1yCO2_{chp} = \sum_{1yr} CO2_{chp}(k)$;

Table S18. A strategy of using mCHP electricity and waste heat for a single household application.

<u>Sub_mode (combustion mode):</u>

Input: mCHP power output W_{chp}

mCHP waste heat $\dot{E}_{chp_{whr}}$

Home power demand W_{h_ee}

Home thermal energy demand $\dot{E}_{h\_ng}$

Outputs: mCHP command ε, grid demand W_{grid}, waste heat loss $\dot{E}_{waste}$

If W_{chp}>W_{h_ee} **then**
  $\varepsilon' = W_{h\_ee}/W_{chp}$;
  If $\varepsilon'\dot{E}_{chp_{whr}}$>$\dot{E}_{h\_ng}$ **then**
    mCHP command $\varepsilon = W_{h\_ee}/W_{chp}$;
    Grid demand $W_{grid} = 0$;
    Waste heat loss $\dot{E}_{waste} = \varepsilon\dot{E}_{chp_{whr}} - \dot{E}_{h\_ng}$;
  Else
    If $W_{chp} - W_{h\_ee} + \dot{E}_{chp_{whr}} > \dot{E}_{h\_ng}$ **then**
      mCHP command $\varepsilon = \left(W_{h\_ee} + \dot{E}_{h\_ee}\right)/\left(W_{chp} + \dot{E}_{chp_{whr}}\right)$;
      Grid demand $W_{grid} = 0$;
      Waste heat loss $\dot{E}_{waste} = 0$;
    Else
      mCHP command $\varepsilon = 1$;
      Grid demand $W_{grid} = \dot{E}_{h\_ng} - (W_{chp} - W_{h\_ee} + \dot{E}_{chp_{whr}})$;
      Waste heat loss $\dot{E}_{waste} = 0$;
    End if
  End if
Else
  mCHP command $\varepsilon = 1$;
  If $\dot{E}_{chp_{whr}}$>E_{h_ng} **then**
    Grid demand $W_{grid} = W_{h\_ng} - W_{chp}$;
    Waste heat loss $\dot{E}_{waste} = \dot{E}_{chp_{whr}} - \dot{E}_{h\_ng}$;
  Else
    Grid demand $W_{grid} = (W_{h\_ee} - W_{chp}) + (\dot{E}_{h\_ng} - \dot{E}_{chp\_whr})$;
    Waste heat loss $\dot{E}_{waste} = 0$;
  End if
End if

Supplemental Note 19: Decarbonization and cost savings

The 10 single-family homes listed in Supplemental Note 17 were studied to evaluate the cost savings and decarbonization of the proposed mCHP with the operation strategy shown in Supplemental Note 18. The results are shown in Table S19. Figures S14(a) and S14(b) show the effect of mCHP applications on annual decarbonization and cost savings of the 10 households in different climate zones. Figures S15(a) and S15(b) show the natural gas energy consumption and waste heat loss of mCHP applications in the 10 households.

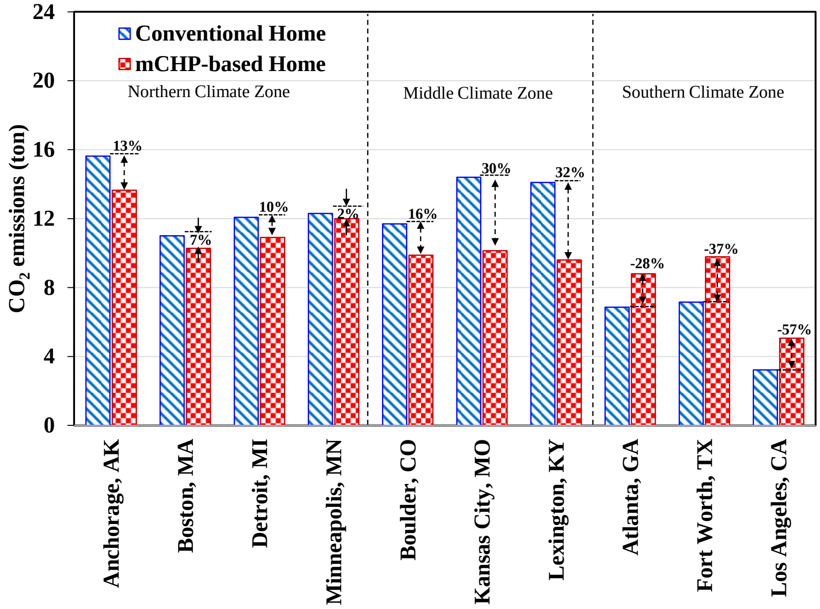

(a) CO_2

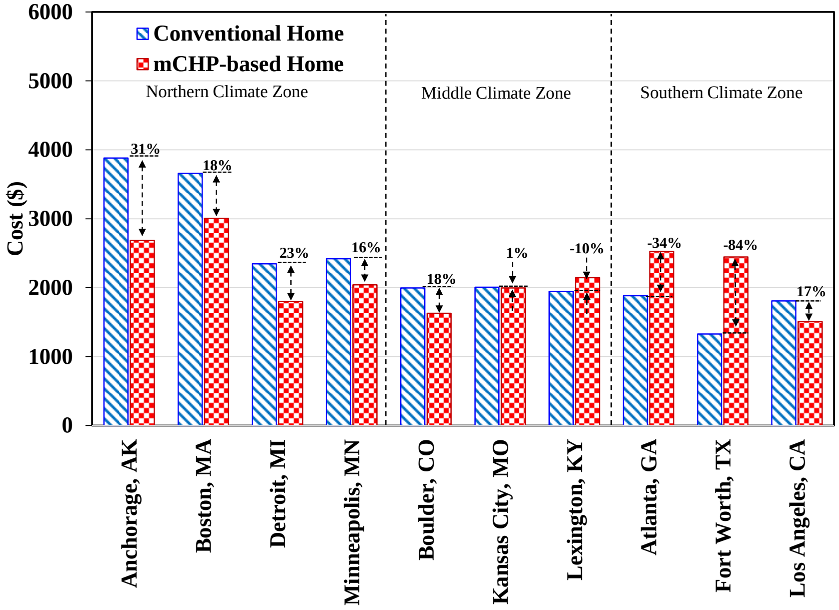

(b) Cost

Figure S14. Effect of mCHP applications on annual (a) decarbonization and (b) cost savings of the 10 households.

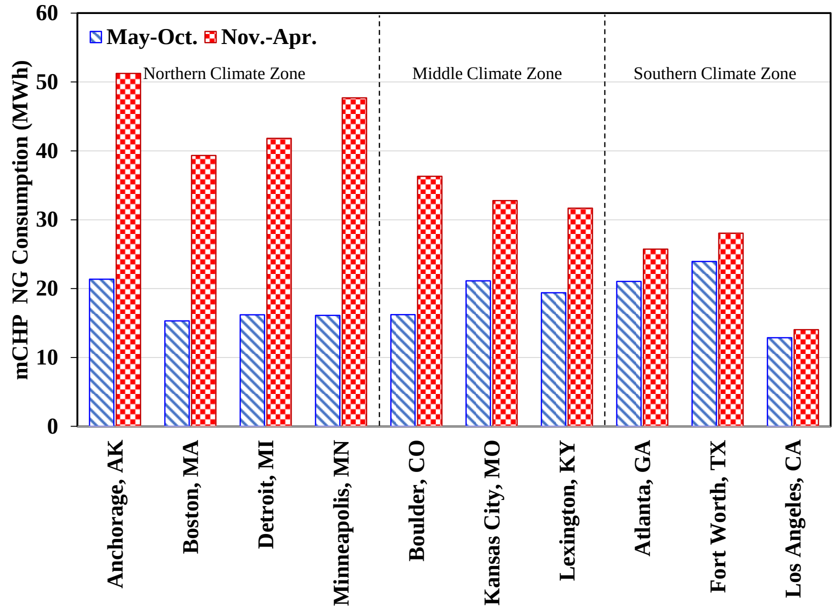

(a) Energy consumption

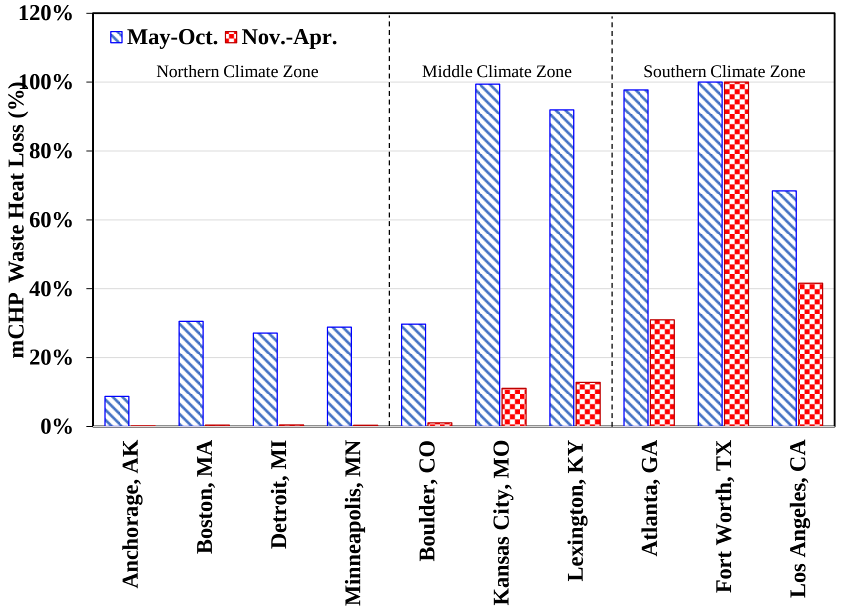

(b) Waste heat loss

Figure S15. The (a) natural gas energy consumption and (b) waste heat loss of mCHP applications in the 10 households.

Table S19. Summary of the decarbonization and cost savings of mCHP applications in the selected 10 households

Location	Home						CHP									
	May–Oct. electricity use (MWh)	Nov.–Apr. electricity use (MWh)	May–Oct. NG use (MWh)	Nov.–Apr. NG use (MWh)	1-year CO_2e (ton)	1-year cost ($)	May–Oct. NG use (MWh)	Nov.–Apr. NG use (MWh)	May–Oct. waste heat loss (MWh)	Nov.–Apr. waste heat loss (MWh)	1-year CO_2e (ton)	1-year cost ($)	1-year CO_2e savings %	1-year cost savings %	Life span CO_2 savings (ton)	Life span cost savings ($)
Anchorage, AK	3.97	5.34	13.72	41.30	15.63	3,881	21.36	51.24	8.8%	0.2%	13.65	2,686	12.6%	30.8%	39.48	35,971
Boston, MA	3.90	5.04	6.59	30.34	11.00	3,657	15.32	39.33	30.5%	0.4%	10.27	3,006	6.6%	17.8%	14.49	21,396
Detroit, MI	3.93	5.10	7.49	32.60	12.07	2,346	16.20	41.82	27.1%	0.4%	10.91	1,799	9.7%	23.3%	23.30	18,591
Minneapolis, MN	3.95	5.25	7.23	38.01	12.30	2,420	16.10	47.69	28.9%	0.4%	12.00	2,042	2.5%	15.6%	6.12	14,057
Boulder, CO	3.97	5.04	7.23	27.32	11.69	1,996	16.23	36.30	29.7%	1.1%	9.88	1,629	15.5%	18.4%	36.27	13,755
Kansas City, MO	6.40	6.49	0.68	19.98	14.40	2,007	21.14	32.79	99.4%	11.1%	10.14	1,995	29.6%	0.6%	85.26	4,206
Lexington, KY	5.86	6.45	0.90	18.76	14.09	1,947	19.40	31.68	91.9%	12.8%	9.60	2,146	31.8%	−10.2%	89.77	−1,440
Atlanta, GA	6.42	6.26	0.26	11.29	6.86	1,883	21.05	25.72	97.7%	31.0%	8.79	2,526	−28.3%	−34.1%	−38.75	−13,388
Fort Worth, TX	7.30	8.58	0.00	0.00	7.16	1,328	23.93	28.04	100%	100%	9.78	2,445	−36.7%	−84.1%	−52.57	−29,485
Los Angeles, CA	3.93	3.93	2.22	4.81	3.22	1,808	12.88	14.04	68.4%	41.6%	5.06	1,507	−57.4%	16.6%	−36.89	11,960

Northern zone: Anchorage, AK; Boston, MA; Detroit, MI; Minneapolis, MN.
Middle zone: Boulder, CO; Kansas City, MO; Lexington, KY.
Southern zone: Atlanta, GA; Fort Worth, TX; Los Angeles, CA.

Supplemental Note 20: The novelty of the mCHP system

It is always attractive, important, and challenging to achieve higher fuel-to-electricity efficiency and cost effectiveness in the development of mCHPs. The fuel-to-electricity efficiency for a conventional mCHP is no more than 30% because of high heat loss to ambient air with the higher surface area/volume ratio of small engines [30]. The reported mCHP prototype enables up to 35.2% of fuel-to-electricity efficiency and nearly 93% of the overall mCHP efficiencies. Compared with conventional ICEs, the OP4S engine has 60% fewer parts per engine unit and therefore, lowers materials and manufacturing costs. In addition, it enables better mCHP reliability and durability, indicating a longer life span. Consequently, the combination of high efficiency and reasonable cost offers significant potential deployment and market penetration opportunities for the reported mCHP in the US residential sector. The mCHP prototype based on the OP4S engine also can be powered by traditional fuels such as natural gas or propane and also has the potential to run carbon-free fuels like hydrogen. This feature indicates that the technology has substantial potential to support the transition from current conventional fossil fuels to zero carbon emissions in the future. In addition, as shown in Figure 1, the mCHP is a compact and portable device, allowing high versatility for installation locations.

Supplemental References

1. EC Power. XRGI CHP, https://www.ecpower.eu/en/xrgi-systems.html; [accessed February 28, 2024].
2. Barbieri, E. S., Spina, P. R. & Venturini, M. Analysis of innovative micro-CHP systems to meet household energy demands. *Appl. Energy* **97**, 723–733 (2012).
3. Taie, Z., West, B., Szybist, J., Edwards, D., Thomas, J., Huff, S., Vishwanathan, G. & Hagen, C. Detailed thermodynamic investigation of an ICE-driven, natural gas-fueled, 1 kWe micro-CHP generator. *Energy Convers. Manag.* **166**, 663–673 (2018).
4. De Paepe, M., D'Herdt, P. & Mertens, D. Micro-CHP systems for residential applications. *Energy Convers. Manag.* **47**(18–19), 3435–3446 (2006).
5. Taie, Z. & Hagen, C. Experimental thermodynamic first and second law analysis of a variable output 1–4.5 kWe, ICE-driven, natural-gas fueled micro-CHP generator. *Energy Convers. Manag.* **180**, 292–301 (2019).
6. Thomas, B. Benchmark testing of Micro-CHP units. *Appl. Therm. Eng.* **28**(16), 2049–2054 (2008).
7. Axiom Energy Group. Micro-combined heat and power, https://www.axiom-energy.com/microchp; [accessed February 28, 2024].
8. Glenergy. Dachs—Mini CHP Combined Heat and Power Technical Specification, http://glenergy.ie/wp-content/uploads/2016/10/Dachs_-_Mini_CHP_Technical_specification.pdf, 2016 [accessed February 28, 2024].
9. TOTEM 10 Datasheet. https://www.totem.energy/wp-content/uploads/Scheda-tecnica_TOTEM-10_ENG.pdf; [accessed February 28, 2024].
10. Yanmar. Combined Heat and Power (CHP), https://www.yanmarenergysystems.com/chp/; [accessed February 28, 2024].
11. Kim, C. K. & Yoon, J. Y. Performance analysis of bladeless jet propulsion micro-steam turbine for micro-CHP (combined heat and power) systems utilizing low-grade heat sources. *Energy* **101**, 411–420 (2016).
12. SOLO Stirling 161. https://www.scribd.com/document/353312261/SOLO-Stirling-161; [accessed February 28, 2024].
13. Bell, M., Swinton, M., Entchev, E., Gusdorf, J., Kalbfleisch, W., Marchand, R. & Szadkowski, F. Development of micro combined heat and power technology assessment capability at the Canadian Centre for Housing Technology. B-6010. Ottawa, Ontario, Canada. (2003).
14. Qiu, G., Shao, Y., Li, J., Liu, H. & Riffat S. B. Experimental investigation of a biomass-fired ORC-based micro-CHP for domestic applications. *Fuel* **96**, 374–382 (2012).
15. Qiu, K. & Hayden, A. C. S. Implementation of a TPV integrated boiler for micro-CHP in residential buildings. *Appl. Energy* **134**, 143–149 (2014).
16. Redox Power Systems. https://www.redoxenergy.com/docs/Redox%20Cube%20Datasheet.pdf, [accessed February 28, 2024].
17. WATT Imperium. https://www.wattfuelcell.com/portable-power/watt-imperium/; [accessed February 28, 2024].
18. Hu, H., Wu, Y., Batou, A. & Ouyang, H. Uncertainty propagation with B-spline based interval field decomposition method in boundary value problems. *Appl. Math. Model.* **123**, 159–177 (2023).

19. Gao, Z., LaClair, T. J., Nawaz, K., Wu, G., Hao, P., Boriboonsomsin, K., Todd, M., Barth, M. & Goodarzi, A. Comprehensive powertrain modelling for heavy-duty applications: A study of plug-in hybrid electric bus. *Energy Convers. Manag.* **252**, 115071 (2022).

20. Moran, M. J., Shapiro, H. N., Boettner, D. D. & Bailey, M. B. Fundamentals of Engineering Thermodynamics. John Wiley & Sons. (2010).

21. Sayin, C. E. N. K., Hosoz, M., Canakci, M. & Kilicaslan, I. Energy and exergy analyses of a gasoline engine. *Int. J. Energy Res.* **31**(3), 259–273 (2007).

22. Choose Energy. Electricity Generation by State, https://www.chooseenergy.com/data-center/electricity-sources-by-state/; [accessed February 28, 2024].

23. US Energy Information Administration. How much carbon dioxide is produced per kilowatt-hour of U.S. electricity generation? https://www.eia.gov/tools/faqs/faq.php?id=74&t=11#:~:text=In%202019%2C%20total%20U.S.%20electricity,of%20CO2%20emissions%20per%20kWh; [accessed February 28, 2024].

24. US Energy Information Administration. US Electricity Profile 2021, https://www.eia.gov/electricity/state/archive/2020/; [accessed February 28, 2024].

25. R7: US Energy Information Administration. Natural Gas Prices 2021. https://www.eia.gov/dnav/ng/ng_pri_sum_a_EPG0_PRS_DMcf_a.htm; [accessed February 28, 2024].

26. EnergyPlus. https://energyplus.net/; [accessed February 28, 2024].

27. Sargent, M. R., Floerchinger, C., McKain, K., Budney, J., Gottlieb, E. W., Hutyra, L. R., Rudek, J. & Wofsy, S. C. Majority of US urban natural gas emissions unaccounted for in inventories. *Proc. NAS* **118** (44), e2105804118 (2021).

28. US Energy Information Administration. How much electricity is lost in electricity transmission and distribution in the United States? https://www.eia.gov/tools/faqs/faq.php?id=105&t=3; [accessed February 28, 2024].

29. US Energy Information Administration. Carbon dioxide emissions coefficients by fuel, https://www.eia.gov/environment/emissions/co2_vol_mass.php; [accessed February 28, 2024].

30. Ausserer, J. K., Litke, P. J., Groenewegen, J. R., Rowton, A., Polanka, M. & Grinstead, K. Development of test bench and characterization of performance in small internal combustion engines. *SAE Technical Paper* **2013-32-9036**, (2013).
